# ZNF643/ZFP69B Exerts Oncogenic Properties and Associates with Cell Adhesion and Immune Processes

**DOI:** 10.3390/ijms242216380

**Published:** 2023-11-15

**Authors:** Urszula Oleksiewicz, Marta Machnik, Joanna Sobocińska, Sara Molenda, Anna Olechnowicz, Anna Florczak, Julia Mierzejewska, Dominika Adamczak, Mikołaj Smolibowski, Mariusz Kaczmarek, Andrzej Mackiewicz

**Affiliations:** 1 Department of Cancer Immunology, Chair of Medical Biotechnology, Poznan University of Medical Sciences, 8 Rokietnicka Street, 60-806 Poznan, Poland; oleksiewiczu@ump.edu.pl (U.O.);; 2 Department of Diagnostics and Cancer Immunology, Greater Poland Cancer Center, 15 Garbary Street, 61-866 Poznan, Poland; 3 Doctoral School, Poznan University of Medical Sciences, 70 Bukowska Street, 60-812 Poznan, Poland; 4 Department of Histology and Embryology, Poznan University of Medical Sciences, 6 Święcickiego Street, 60-781 Poznan, Poland

**Keywords:** ZNF643, ZFP69B, KRAB-ZFP, oncogenes, transcriptomic profiling, ChIP-seq, TCGA datasets

## Abstract

The global cancer burden remains high; thus, a better understanding of the molecular mechanisms driving carcinogenesis is needed to improve current prevention and treatment options. We previously detected the ZNF643/ZFP69B gene upregulated in multiple tumors, and we speculated it may play a role in tumor biology. To test this hypothesis, we employed TCGA-centered databases to correlate ZNF643 status with various clinicopathological parameters. We also performed RNA-seq analysis and in vitro studies assessing cancer cell phenotypes, and we searched for ZNF643-bound genomic loci. Our data indicated higher levels of ZNF643 in most analyzed tumors compared to normal samples, possibly due to copy number variations. ZNF643 mRNA correlated with diverse molecular and immune subtypes and clinicopathological features (tumor stage, grade, patient survival). RNA-seq analysis revealed that ZNF643 silencing triggers the deregulation of the genes implicated in various cancer-related processes, such as growth, adhesion, and immune system. Moreover, we observed that ZNF643 positively influences cell cycle, migration, and invasion. Finally, our ChIP-seq analysis indicated that the genes associated with ZNF643 binding are linked to adhesion and immune signaling. In conclusion, our data confirm the oncogenic properties of ZNF643 and pinpoint its impact on cell adhesion and immune processes.

## 1. Introduction

Despite enormous efforts and constant progress in modern medicine, the global cancer burden remains high and is envisaged to increase in the following years [1]. This complex disease is characterized by high intra-tumor and intra-patient heterogeneity. Hence, the treatment is challenging, and the risk of failure and disease relapse is still very high. Cancer cells are deregulated by genetic mutations, changes in epigenetic processes, and chromosomal instability [2,3]. Altogether, many biological processes of normal cells adapt to sustain a high proliferation rate, avoid apoptotic signals and immune system control, and gain the potential to metastasize. These adaptations lead to clonal advantage, which allows the disease to develop and spread across the body. Knowing the specific mechanisms guiding cancer-related alterations is yet of high importance.

Krüppel-associated box domain zinc finger proteins (KRAB-ZFPs) are the most prominent family of transcription factors in the human genome, yet many proteins remain poorly characterized. KRAB-ZFPs may act as potent repressors of gene transcription by directly binding to specific DNA sequences. The binding is mediated by C-terminus zinc finger motifs (C2H2), whereas the N-terminus is involved in TRIM28 binding via the KRAB domain [4]. TRIM28 is a scaffold protein for a repressive macromolecular complex. It binds histone methyltransferase (SETDB1) responsible for methylation of H3K9, nucleosome remodeling and deacetylase complex (NuRD), and heterochromatin protein 1 (HP1) [4]. Despite their direct repressive function, many KRAB-ZFPs may also participate in the activation of gene transcription [4,5]. As such, KRAB-ZFPs may be implicated in diverse biological processes, including cancer induction and development [4,5,6].

Our previous studies aimed to explore the transcriptomic signature of KRAB-ZFPs in a pan-cancer setting. Our analysis based on the Cancer Genome Atlas (TCGA) datasets demonstrated that many KRAB-ZFPs are downregulated in various cancer types. In contrast, only a small portion becomes overexpressed [7]. One of the identified overexpressed KRAB-ZFP factors was Zinc Finger Protein 643 (ZNF643), also known as ZFP69B. Our results indicated that ZNF643 was upregulated in 13 out of 16 tumor types compared to normal samples. Using a single-gene approach, we experimentally validated its increased level in the independent panels of lung and breast cancer tissues and cell lines. Furthermore, we showed that the ZNF643 mRNA level differs among molecular subtypes in lung adenocarcinoma (LUAD) and breast cancer (BRCA). We also found that high ZNF643 was related to shorter overall survival in squamous cell lung carcinoma (LUSC) and BRCA patients [7]. These observations implied hypothetical pro-tumorigenic features of ZNF643.

ZNF643 belongs to the KRAB-ZFP proteins, harboring an additional SCAN (SRE-ZBP, CTfin51, AW-1, and Number18 cDNA) domain that may mediate protein–protein interactions [6]. The exact biological function of ZNF643 remains insufficiently characterized. However, it was shown to be associated with ferroptosis [8], a type of nonapoptotic, iron-dependent cell death resulting from lowered cystine uptake that leads to reduced antioxidant defense and the generation of lethal lipid reactive oxygen species [9]. ZNF643 expression demonstrated a two-fold increase in the transcriptomic profiling of the HT-1080 fibrosarcoma cell line treated with erastin, an inducer of ferroptosis [8]. Interestingly, recently published data identified *ZNF643* as one of the ferroptosis-related genes (FRGs) with a prognostic value for hepatocellular carcinoma [10,11], breast cancer [12], and colon cancer patients [13]. In addition, Wang and colleagues provided evidence that liver cancer cells with knocked-down expression of ZNF643 demonstrate decreased proliferation, migration, and invasion and increased erastin-induced ferroptosis [10]. These results suggest the oncogenic properties of ZNF643, at least in liver cancer [10].

In the current study, we aimed to extend our former pan-cancer research [7] and test the influence of ZNF643 on cancer cell transcriptome and behavior. Here, we demonstrate that ZNF643 is upregulated in most of the tumor types available in TCGA, and copy number variations (CNVs) seem to be implicated in altered ZNF643 expression. Its mRNA levels are correlated with various clinicopathological parameters, including patient survival, tumor stage, grade, molecular, and immune subtyping. Although the influence of ZNF643 on cellular transcriptome and phenotype was cell-type dependent, it was associated with several cancer-related processes, such as growth, adhesion, and immune system. Specifically, our experimental data indicated that ZNF643 may positively correlate with migration and invasion. Finally, we identified genes associated with ZNF643 binding sites, including those participating in cell adhesion and immune signaling. Altogether, our results confirm the oncogenic potential of ZNF643 and highlight its implications in cellular adhesion and immune-related processes.

## 2. Results

### 2.1. Association between ZNF643 Expression and Clinical Parameters in a Pan-Cancer Setting

**Figure 1:**
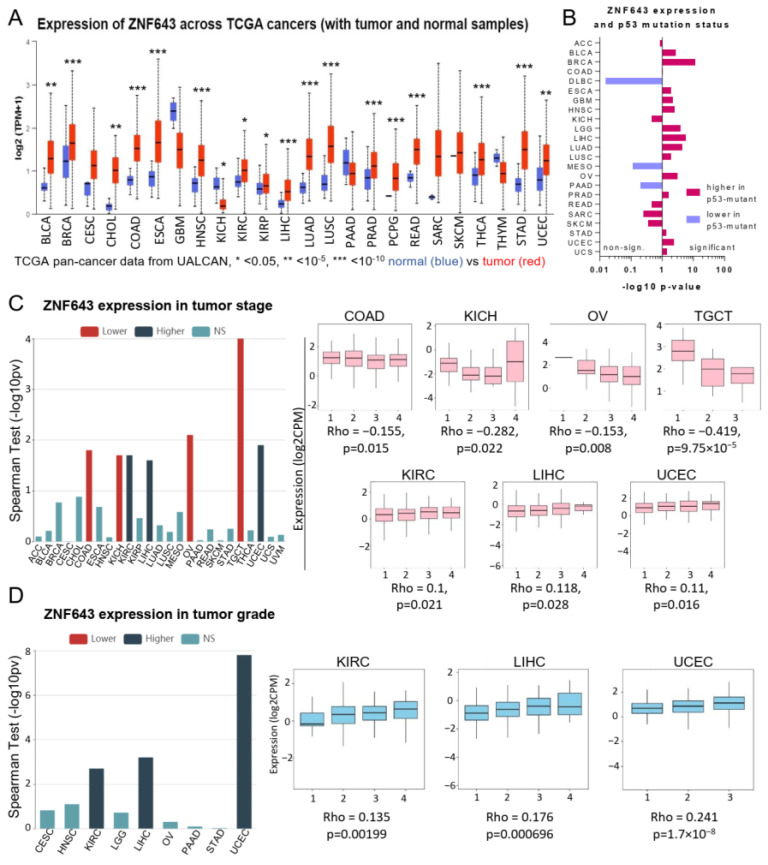
ZNF643 status in TCGA samples. (**A**) ZNF643 expression in normal (blue) and tumor (red) samples among distinct TCGA cohorts assessed with the UALCAN database. Statistical significance was calculated based on the student *t*-test. (**B**) Association between ZNF643 gene expression and p53 mutation status. Spearman’s correlation between ZNF643 gene expression and (**C**) tumor stage and (**D**) tumor grade. The data was downloaded from TISIDB database. The graphs on the left-hand side represent the *p*-values (transformed into –log10 *p*-values) as assessed with Spearman correlation test (NS: non-significant). Higher ZNF643 expression was observed in the tumors with lower (red) or higher (dark blue) (**C**) or grade (**D**). The tumors presenting statistically significant correlations are additionally shown on the barplots on the right. The scale from 1 to 4 on the X axis (**C**,**D**) denotes, respectively: the lowest and highest stage and grade. Adrenocortical carcinoma (ACC); bladder urothelial carcinoma (BLCA); breast invasive carcinoma (BRCA); cervical squamous cell carcinoma and endocervical adenocarcinoma (CESC); cholangiocarcinoma (CHOL); colon adenocarcinoma (COAD); lymphoid neoplasm diffuse large B-cell lymphoma (DLBC); esophageal carcinoma (ESCA); glioblastoma multiforme (GBM); head and neck squamous cell carcinoma (HNSC); kidney chromophobe (KICH); kidney renal clear cell carcinoma (KIRC); kidney renal papillary cell carcinoma (KIRP); brain lower grade glioma (LGG); liver hepatocellular carcinoma (LIHC); lung adenocarcinoma (LUAD); lung squamous cell carcinoma (LUSC); mesothelioma (MESO); ovarian serous cystadenocarcinoma (OV); pancreatic adenocarcinoma (PAAD); prostate adenocarcinoma (PRAD); pheochromocytoma and paraganglioma (PCPG); rectum adenocarcinoma (READ); sarcoma (SARC); skin cutaneous melanoma (SKCM); stomach adenocarcinoma (STAD); testicular germ cell tumors (TGCT); thyroid carcinoma (THCA); thymoma (THYM); uterine corpus endometrial carcinoma (UCEC); uterine carcinosarcoma (UCS); uveal melanoma (UVM).

Our previous data showed that ZNF643 expression is commonly upregulated in 16 tumor types compared to normal samples [7]. Here, we confirmed and extended this observation to other cancers using the UALCAN database. The data revealed that ZNF643 mRNA is significantly elevated in 17 out of 24 analyzed tumors (Figure 1A) and decreased only in kidney chromophobe (KICH). The higher expression could be noted for the cancers of the bladder (BLCA), breast (BRCA), colon (COAD), esophagus (ESCA), head and neck (HNSC), kidney (KIRC, KIRP), liver (LIHC), lung (LUAD, LUSC), prostate (PRAD), rectum (READ), thyroid (THCA), stomach (STAD), and uterine (UCEC). Also, cholangiocarcinoma (CHOL), and pheochromocytoma, and paraganglioma (PCPG) showed increased expression of ZNF643. Moreover, we noted a significantly higher ZNF643 expression in the samples with mutated p53 in 14 tumor types (BLCA, BRCA, ESCA, glioblastoma multiforme (GBM), low-grade glioblastoma (LGG), LIHC, LUAD, LUSC, ovarian cancer (OV), STAD, UCEC, and uterine carcinosarcoma (UCS)) (Figure 1B). ZNF643 expression levels varied as well among different molecular subtypes in the case of BRCA, COAD, LGG, LIHC, LUSC, OV, PCPG, and STAD (Figure S1). Furthermore, we observed a negative correlation between ZNF643 expression and tumor stage in COAD, KICH, OV, and testicular germ cell tumors (TGCT) (Figure 1C). In contrast, a positive correlation was noticed between ZNF643 mRNA level and tumor stage and grade in KIRC, LIHC, and UCEC (Figure 1C,D).

**Figure 2:**
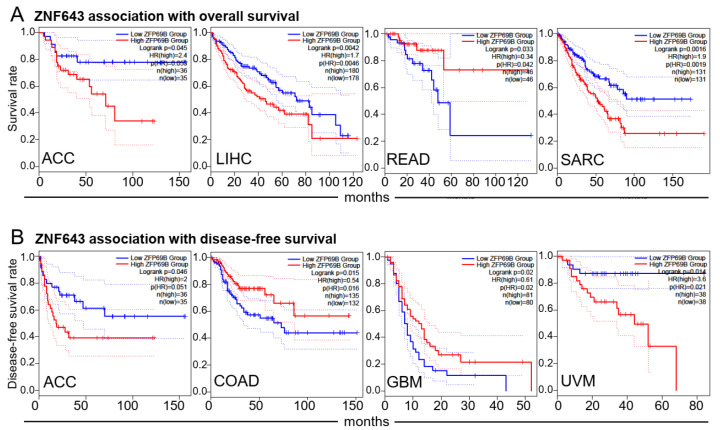
ZNF643 expression and patient prognosis. (**A**,**B**) In several analyzed tumors, high and low-risk groups of patients present differences in ZNF643 median expression as assessed with the log-rank tests of overall survival (**A**) and disease-free survival (**B**). Presented graphs with the Kaplan–Meier curves contain information about the numbers of patients in high (red) and low (blue) ZNF643 expression groups, calculated hazard ratios (HR), and *p*-values. Dotted lines represent 95% Confidence Interval range.

Finally, we probed the prognostic potential of ZNF643 expression (Figure 2). High ZNF643 mRNA was associated with poorer overall survival (OS) and disease-free survival (DSF) in adrenocortical carcinoma (ACC), OS in LIHC and sarcoma (SARC), and DFS in uveal melanoma (UVM). On the contrary, lower ZNF643 expression was linked to worse OS in READ and DFS in COAD and GBM (Figure 2).

### 2.2. ZNF643 Expression and Tumor Immune Status

**Figure 3:**
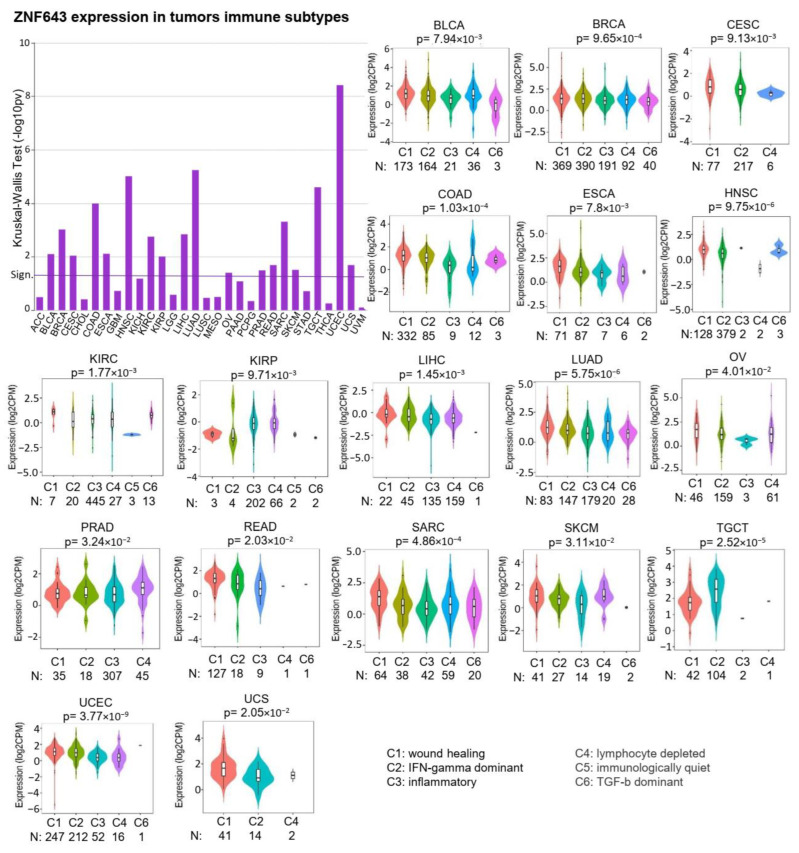
ZNF643 association with tumor immune subtypes. Distinct immune subtypes (C1–C6) of analyzed TCGA tumors represent differences (Kruskal–Wallis test) in ZNF643 gene expression. Transformed *p*-values (−log10) are present on a barplot. Violin plots represent tumors with statistically significant expression differences, showing *p*-value calculated with the Kruskal–Wallis test. The number of samples within different immune subtypes is displayed below plots.

**Figure 4:**
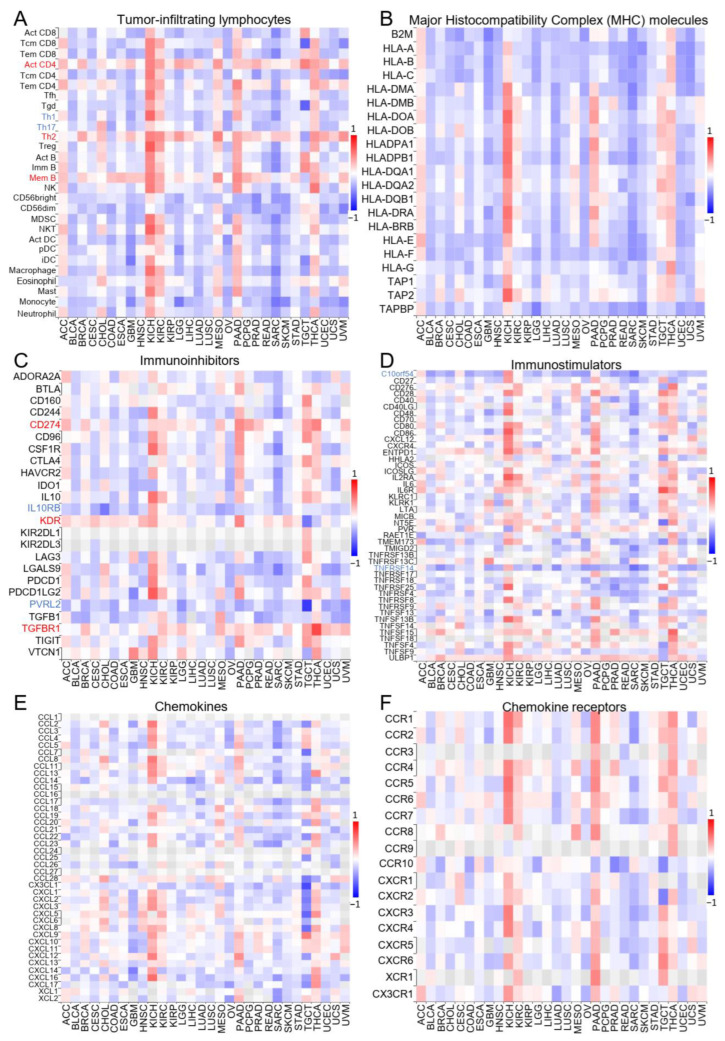
ZNF643 correlation with tumor immunology. (**A**–**F**) The heatmaps displaying the Spearman’s correlation rho values between the expression level of ZNF643 and the expression of the genes specific for tumor-infiltrating lymphocytes (**A**), MHC molecules (**B**), immunoinhibitors (**C**), immunostimulators (**D**), chemokines (**E**), and chemokine receptors (**F**). Blue boxes and fonts represent negative correlation, while red—positive correlation.

We further used the TISIDB database to investigate the association between ZNF643 expression and tumor immune signatures. First, we observed that in 18 out of 30 tumors, ZNF643 expression differed significantly across six immune subtypes (Figure 3) defined by Thorsson and colleagues [14]. In general, higher ZNF643 levels were noted in the wound healing subtype (C1). In contrast, lower expression was frequently found in the inflammatory subtype (C3) and, to a certain extent, in the lymphocyte-depleted subtype (C4). An opposite effect was observed only in KIRP. Compared to the inflammatory subtype, the wound healing subtype is characterized by a lower lymphocyte T helper 1 to 2 ratio (Th1:Th2) and low Th17 [14]. Indeed, in most tumors, ZNF643 expression is positively correlated with the Th2 population and negatively with the Th1 and Th17 content (Figure 4A). Besides the correlation with active lymphocyte T CD4^+^ (Act CD4), ZNF643 correlated with memory lymphocyte B (Mem B) cells. Such a tumor-infiltrating lymphocyte (TIL) signature suggests an association between ZNF643 expression and the humoral response within the tumor mass.

In most of the cancers, ZNF643 expression showed a negative correlation with the remaining TILs, as well as immune-related molecules, such as MHC, immunoinhibitors, immunostimulators, chemokines, and chemokine receptors (Figure 4B–F). Nevertheless, an evident positive correlation between lymphocyte population and immune molecule expression was observed in the case of ACC, KICH, KIRC, MESO, PAAD, and THCA (Figure 4A). The pan-cancer perspective on immune-related molecules was less apparent. ZNF643 correlated positively with CD274 coding for programmed death ligand 1 (PD-L1), vascular endothelial growth factor receptor 2 (KDR), and transforming growth factor beta receptor 1 (TGFBR1) (Figure 4C). A negative association was noted for interleukin 10 receptor subunit beta (IL10RB) and poliovirus receptor-like 2 (PVRL2) immunoinhibitors (Figure 4C), as well as V-domain Ig suppressor of T cell activation (known as chromosome 10 open reading frame 54—C10orf54), and tumor necrosis factor receptor superfamily member 14 (TNFRSF14) immunostimulator (Figure 4D). Altogether, the association between ZNF643 and immune status depended mainly on the type of cancer. In general, our observations indicate higher ZNF643 expression in immunologically cold tumors. Exceptions to this rule are ACC, KICH, KIRC, MESO, PAAD, and THCA.

### 2.3. ZNF643 Expression Is Associated with Copy Number Variations (CNVs) in Many Tumors

**Figure 5:**
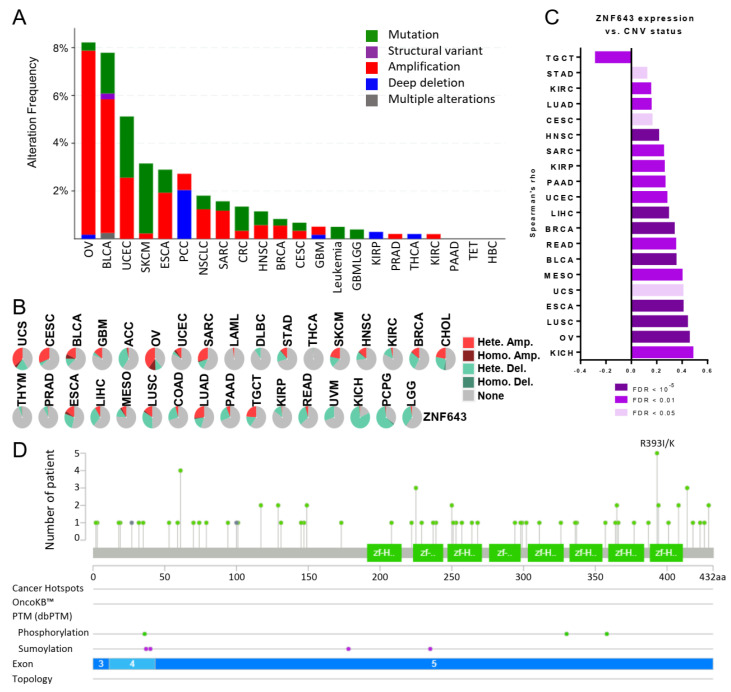
Mutations and structural variations of ZNF643 across TCGA samples. (**A**,**B**) Different tumor types present distinct mutational statuses of *ZNF643*, as evidenced by cbioportal (**A**) and GSCA (**B**) algorithms. For the cbioportal analysis (**A**), we selected the “cancer study” mode with a minimum of 100 samples within each analyzed cohort. Light green lollipops represent missense mutations of unknown significance, and their heights correspond to the total number of patients with a given mutation. Green boxes mark zinc finger double domains (zf-H2C2_2). Green dots represent ZNF643 phosphorylation sites (threonines: 36, 330, and 358), whereas purple dots denote sumoylation sites (lysines: 37, 40, 178, 235). (**C**) ZNF643 gene expression is associated with CNV status as assessed with Spearman correlation analysis. (**D**) Graphical representation of point mutations located within the ZNF643 gene. Abbreviations: pheochromocytoma (PCC), non-small cell lung cancer (NSCLC), colorectal cancer (CRC), glioma (GBMLGG), thyroid epithelial tumor (TET), hepatobiliary cancer (HBC).

Next, we tested whether the *ZNF643* gene may undergo any genetic and epigenetic alterations during carcinogenesis. We analyzed genetic changes using the cbioportal and GSCA databases. According to cbioportal, the overall genetic and genomic variability occurs in 1.9% of all analyzed tumors of various origins, and amplification events seem to be the major cause of these alterations (Figure 5A). The most frequent genetic and genomic alterations were identified in OV and BLCA (~8% of the tumor samples) (Figure 5A). In the GSCA database, CNV events were identified at a higher percentage (Figure 5B) than in the cbioportal database. For example, GSCA algorithms called amplifications in more than half of OV samples and deletions in more than half of KICH and PCPG samples (Figure 5B). Notably, CNV status positively correlated with ZNF643 expression in 19 tumors, while a negative correlation was observed only in the case of TGCT (Figure 5C). Both portals indicated a relatively low number of point mutations (Figure 5A,D). Cbioportal visualization demonstrated evenly distributed mutations across the *ZNF643* gene (Figure 5D). Most point mutations were unique, and only a few were identified in two to five different tumor samples (Figure 5D, Supplementary File S1). The most frequent mutation (identified in five samples) resulted in a missense mutation (R393I/K amino acid change) in the last (8th) zinc finger motif. The variant allele frequency ranged from 20 to 67%. Although the mutation is predicted to have a deleterious effect on protein function, its effect on cell phenotype remains unknown. None of the variants was classified as drivers in the cbioportal database, and their annotations had “unknown” oncogenic status (Supplementary File S1).

We further analyzed the CG methylation profile within the *ZNF643* promoter. The data available on the UALCAN website indicated significant differences between tumor and normal tissues, pinpointing decreased and increased methylation in the primary tumor samples (Figure S2). However, the methylation level in all samples fell within the hypomethylation range (beta values < 0.1). Thus, we concluded that the observed changes may have limited biological significance for ZNF643 expression. In summary, the *ZNF643* promoter has a low methylation level in both normal and tumor samples, so the influence of methylation status on ZNF643 expression is probably negligible. Instead, CNV events are likely to affect ZNF643 expression in tumor samples, as both phenomena correlate with each other.

### 2.4. Transcriptomic Profiling of the Cells with Silenced Expression of ZNF643

**Figure 6:**
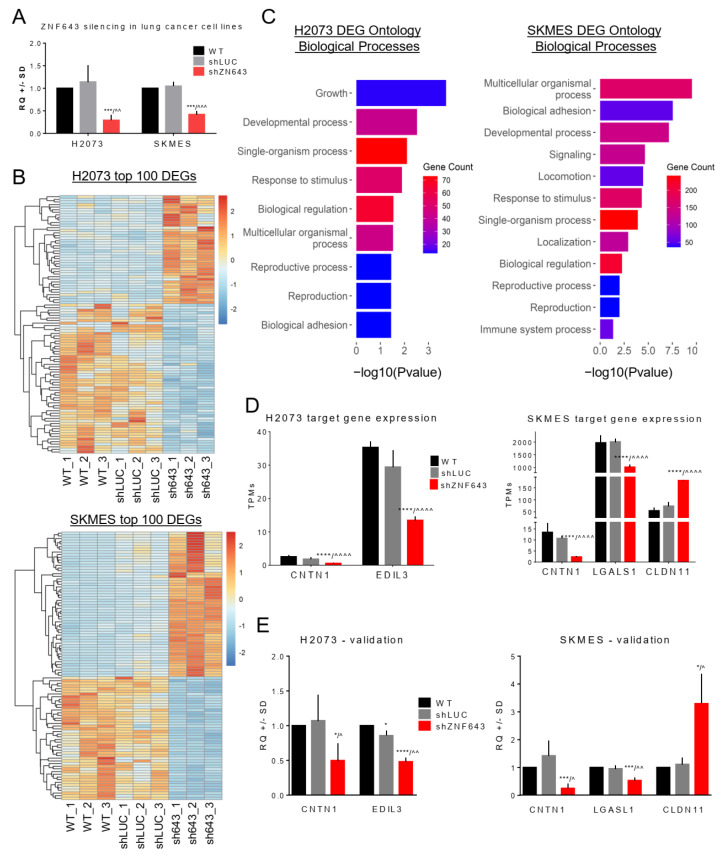
Transcriptomic profile of lung cancer cell lines with decreased expression of ZNF643. **(A**) ZNF643 gene expression level after shRNA-mediated knock-down in H2073 and SKMES cell lines evaluated with qPCR assay relative to ESD internal control. (**B**) Heatmaps representing Z-score normalized TPM (transcript per kilobase per million mapped reads) values and supervised clustering of top 100 (sorted by *p*-value) differentially expressed genes (DEGs) identified between control cells (WT and shLUC) and shZNF643 cells for H2073 (top) and SKMES (bottom). Presented genes met the criteria of cutoff threshold fc ≥ 2 or ≤−2 and *p* < 0.05. (**C**) Barplots depicting enriched biological processes (BP1) connected with identified DEGs for H2073 (left) and SKMES (right). The color scale represents the number of DEGs involved in each process. (**D**) RNA-seq TPM values of normalized expression for selected ZNF643 target genes. (**E**) RT-qPCR analysis of selected target gene expression. The experiment was performed in biological and technical triplicates. *p*-value symbols: * vs. WT; ^ vs. shLUC; */^ *p* < 0.05; ^^ *p* < 0.01; ***/^^^ *p* < 0.001, ****/^^^^ *p* < 0.0001.

While ZNF643 becomes overexpressed in tumor samples, and its level correlates with various clinical parameters, as well as molecular and immune subtypes, we hypothesized that ZNF643 may contribute to oncogenic processes. To test this hypothesis, we first investigated the influence of ZNF643 on the transcriptome of cancer cells. We focused on lung cancer as the most common cancer affecting both sexes worldwide [15]. We used H2073 (lung adenocarcinoma) and SKMES (squamous cell lung cancer) cell lines to knock down ZNF643 expression utilizing an shRNA approach (Figure 6A) and perform RNA-seq analysis. We selected those cell lines based on our previous experiments [7]. Our data indicated ZNF643 overexpression in H2073 and SKMES compared to normal human bronchial epithelium. Moreover, by exploring the depmap portal, we found that H2073 has 3 copies, while SKMES has 4 copies of the *ZNF643* gene. In addition, both cell lines harbor likely loss-of-function mutations in the *TP53* gene (Supplementary File S2). It is possible that these molecular events (CNV, *TP53* mutations) may be implicated in the increased expression of ZNF643 in those cell lines.

In our RNA-seq analyses, we were looking for differentially expressed genes (DEGs) that were significantly deregulated compared to both controls used in the study (WT and shLUC). We identified 46 up- and 62 downregulated genes in the shZNF643 H2073 cell line and 154 up- and 216 downregulated in the shZNF643 SKMES cell line (Figure 6B). DEGs were subjected to Gene Ontology analysis in the DAVID database. Biological Processes (BP1) that were significantly affected in the case of both cell lines included developmental processes, single- and multicellular organism processes, response to stimulus, biological regulation, reproductive process, and biological adhesion (Figure 6C, Figures S3A and S4A). Previously, *ZNF643* was characterized as a gene involved in ferroptosis [8,10,11,12,13]. We did not find ferroptosis among the significantly affected biological processes in our analysis. Yet, several ferroptosis-related genes (FRGs) were deregulated in lung cancer cells after ZNF643 knock-down including transcription factor AP-2-alpha (TFAP2A) in H2073, cyclic AMP-dependent transcription factor (ATF3), cyclin-dependent kinase 1 (CDKN1), solute carrier family 2, and facilitated glucose transporter member 3 (SLC2A3) in SKMES (Supplementary Table S1).

Apart from Staphylococcus aureus infection, identified KEGG pathways were different for both cell lines (Figures S3B, S4B and S5A). In the SKMES cell line, KEGG pathways pointed toward changes in cell adhesion molecules, fluid shear stress and atherosclerosis, AGE-RAGE signaling pathway in diabetic complications, and other pathways related to immune system (e.g., leukocyte transendothelial migration, complement, and coagulation cascades) (Figures S4B and S5A). In H2073, the pathways were related to various metabolic processes (e.g., biosynthesis of cofactors, retinol metabolism) (Figures S4B and S5A). Moreover, the Cellular Component (CC1) mostly perturbed by ZNF643 knock-down in both cell lines was the extracellular region part (Figures S3C, S4C and S5B). No other Cellular Components were identified for H2073. However, for the SKMES cell line, the analysis pinpointed membrane, synapse, and cell junction (Figures S4C and S5B).

Based on these observations, we focused on a few genes implicated in cell adhesion, including oncogenic contactin 1 (CNTN1) [16,17,18], galectin 1 (LGALS1) [19,20,21,22], and epidermal growth factor-like repeats and discoidin I-like domain 3 (EDIL3) [23,24], as well as tumor suppressive claudin 11 (CLDN11) [25,26,27]. CNTN1 was the only gene associated with cell adhesion that was deregulated in both analyzed cell lines, and the RNA-seq data indicated that ZNF643 silencing leads to its downregulation (Figure 6D). In H2073 shZNF643 cell lines, EDIL3 was downregulated, whereas in SKMES shZNF643 cells, LGALS1 expression decreased, and CLDN11 increased (Figure 6D). We confirmed these observations using a single-gene approach by RT-qPCR analysis (Figure 6E). Merged RNA-seq data identified additional three genes (Figure S6) deregulated in both shZNF643 cell lines, and two were related to immune system. ZNF643 silencing led to the overexpression of complement C4B (C4B) and downregulation of major histocompatibility complex, class II, DR beta 1 (HLA-DRB1) (Figure S6). Altogether, these results further support the notion that ZNF643 may be involved in regulating cell proliferation, migration, invasion, metabolism, and immune response.

### 2.5. In Vitro Studies on ZNF643 Function in Cancer Cells

**Figure 7:**
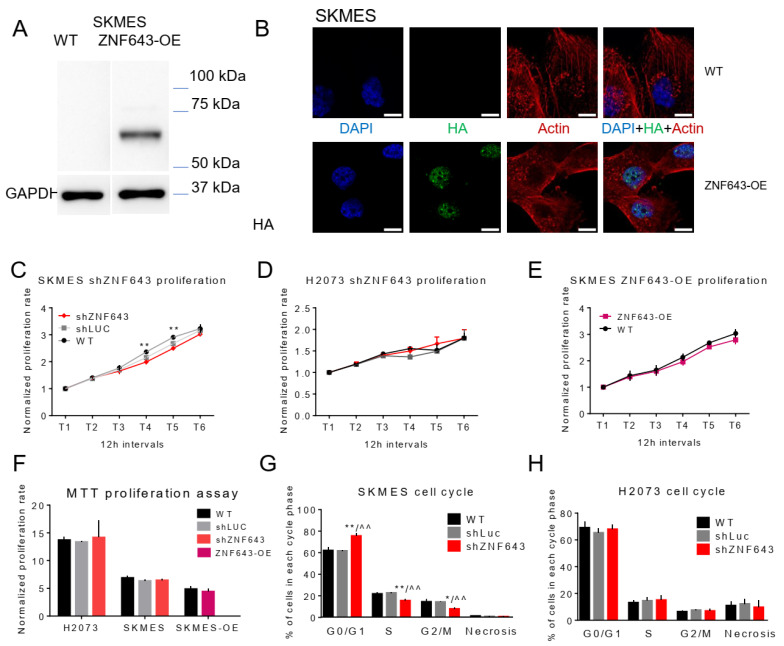
The expression level of ZNF643 is modestly associated with the proliferative potential of cancer cells. (**A**) Western blot analysis assessed the ectopic overexpression of HA-tagged ZNF643 in SKMES lung cancer cell line compared to GAPDH internal control. (**B**) Confocal microscopy confirmed nuclear localization of HA-tagged ZNF643 (green) in SKMES cell line. Actin filament (red) and cell nuclei (blue) are also visualized. The pictures were taken with FV1000 Olympus scanning confocal microscope, 60×. The scale bar represents 10 μm. Cell proliferation of lung cancer cell lines with decreased (shZNF643) and increased (ZNF643-OE) expression of ZNF643 was measured with the real-time proliferation assay on IncuCyte instrument (**C**–**E**) and with MTT colorimetric test (**F**). The analysis of the cell cycle was performed by flow cytometry (**G**,**H**). The bar graphs represent statistics for at least three biological replicates. Statistical significance was calculated with an unpaired t-test. *p*-value symbols: * vs. WT; ^ vs. shLUC; * *p* < 0.05; **/^^ *p* < 0.01.

As transcriptomic profiling revealed that ZNF643 silencing affects the expression of cell adhesion genes with a role in cancer, we moved towards phenotypic studies to test the influence of ZNF643 on lung cancer cell behavior. We created an additional research model besides SKMES and H2073 cell lines with silenced ZNF643, and we overexpressed ZNF643 in SKMES cancer cell lines (Figure 7A). While ZNF643 belongs to the KRAB-ZFP family, whose canonical function is epigenetic repression, we investigated the possibility of nuclear localization of ZNF643. Our ICC data in SKMES (Figure 7B) confirmed that ZNF643 resides mainly in the nucleus, suggesting its potential role as an epigenetic factor. In the nucleus, ZNF643 revealed punctuated staining across a homogenous background, highlighting its increased regional concentration. Faint-punctuated staining was also evident in the cytoplasm (Figure 7B).

**Figure 8:**
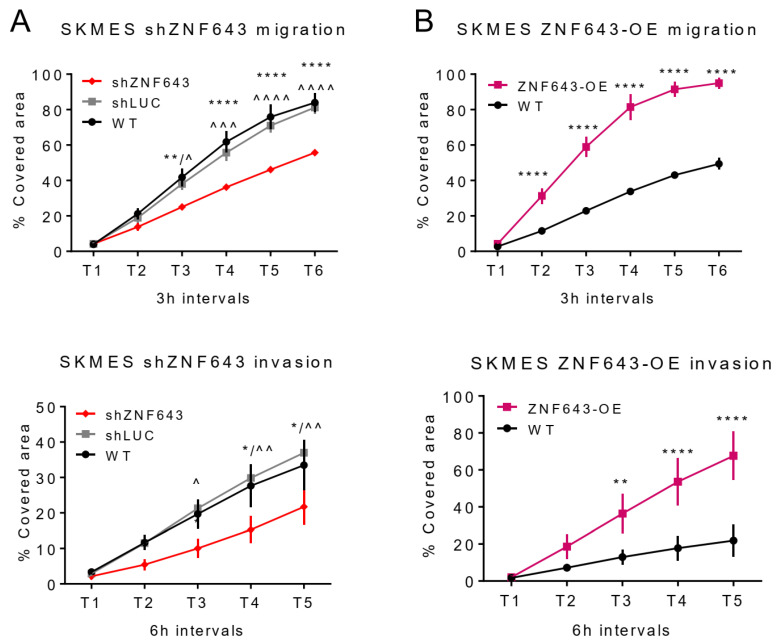
ZNF643 affects the migration and invasiveness of lung cancer cells. A real-time wound healing assay was performed to assess migration (upper panel) and invasion (lower panel) levels in SKMES shZNF643 (**A**), and SKMES ZNF643-OE (**B**). The points represent statistics for at least three biological replicates. Statistical significance was calculated with a two-way ANOVA test corrected with Dunnett’s multiple comparison test. *p*-value symbols: * vs. WT; ^ vs. shLUC; */^ *p* < 0.05; **/^^ *p* < 0.01; ^^^; *p* < 0.001, ****/^^^^ *p* < 0.0001.

These cell line models were further subjected to various phenotypic assays testing cell proliferation, cell cycle, apoptosis, migration, and invasion. Our observations indicated a modest ZNF643 influence on cell proliferation (Figure 7C–F). SKMES shZNF643 cells had a lowered growth rate compared to WT cells, however, only at certain time points (36 and 48 h from the start of the experiment) (Figure 7C). At 48 h post-seeding, SKMES shZNF643 exhibited a small shift towards cell cycle arrest (Figure 7G). The proliferation and cell cycle in the H2073 cell line remained unchanged after ZNF643 knock-down (Figure 7D,F,H). We did not observe any difference in apoptosis for any of the analyzed cell lines (Figure S7). However, we noted a negative correlation between ZNF643 expression level and cell migratory and invasive properties (Figure 8A,B and Figure S8A,B). Upon ZNF643 silencing, the SKMES cells migrated and invaded at a significantly lowered rate compared to WT and shLUC controls (Figure 8A and Figure S8A), while overexpression resulted in an opposite effect (Figure 8B and Figure S8B). Thus, this data indicates that ZNF643 has a negligible influence on proliferation. Moreover, it positively affects cell migration and invasion. Of note, the observed changes in phenotype are cell type dependent.

### 2.6. Identification, Characterization, and Single-Gene Validation of the Targets Bound by ZNF643

**Figure 9:**
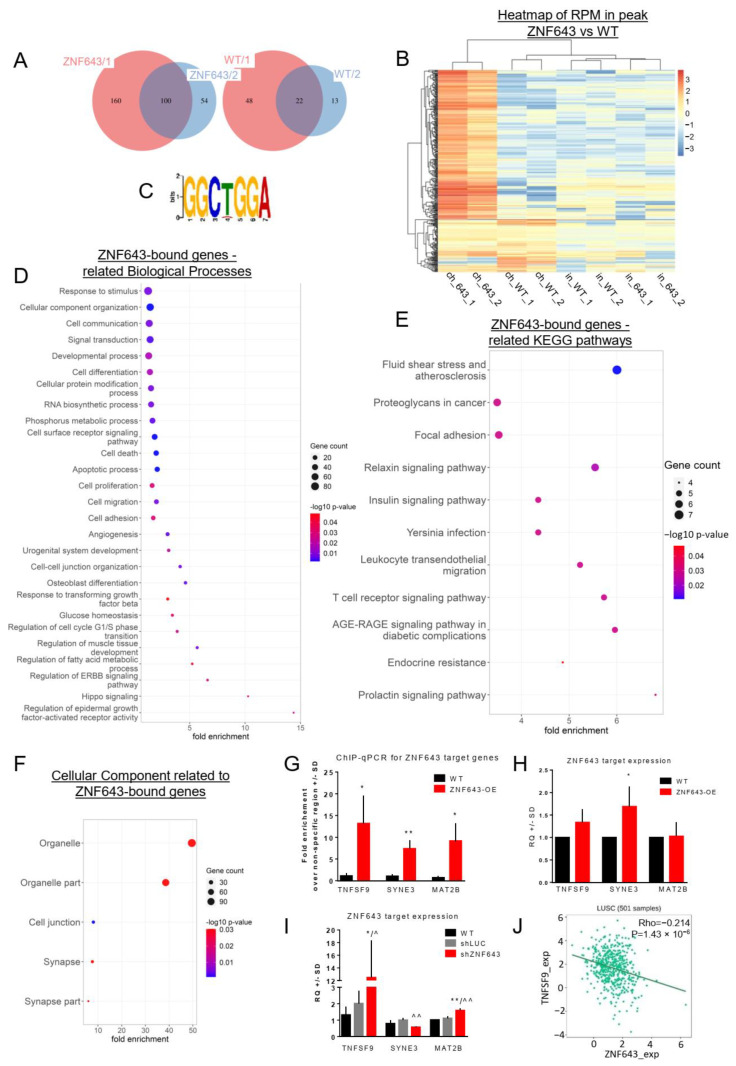
ChIP-seq analysis of genomic regions bound by ZNF643. (**A**) ChIP seq analysis determined the number of SKMES WT and ZNF643-OE differential peaks within two analyzed replicates. (**B**) Heatmap of RPM values in the peaks obtained from ChIP (ch) and input (in) samples of SKMES WT and ZNF643-OE cell variants. The heatmap demonstrates the enrichment pattern of the peaks among other peaks across various samples and in comparison to input. (**C**) A significant short motif sequence detected in the peaks from both replicates. (**D**–**F**) Bubble plots representing enriched Biological Processes (BP ALL) (**D**), KEGG pathways (**E**), and Cellular Components (CC1) (**F**) connected to the genes associated with ZNF643 binding sites. X-axis—fold enrichment; color scale—*p*-value; bubble size—the number of genes involved in each process. (**G**) ChIP-qPCR validation assays for selected target genes associated with ZNF643 binding loci in the SKMES cell line. RT-qPCR assessed the expression level of selected ZNF643 target genes in (**H**) ZNF643-OE and (**I**) shZNF643 cells. The experiment was performed in biological and technical triplicates. Statistical significance was calculated with an unpaired t-test. */^ *p* ≤ 0.05; **/^^ *p* ≤ 0.01; * vs. WT, ^ vs. shLUC; (**J**) Spearman correlation test between TNFSF9 and ZNF643 expression in LUSC samples downloaded from TISIDB database.

To explore the molecular mechanisms behind the ZNF643 function, we sequenced ZNF643-bound DNA from the SKMES cell line (Figure 9 and Figures S9–S11). Our ChIP-seq analysis identified 260 and 154 peaks separately for each of the replicates, and 100 shared peaks were differentially enriched in comparison to WT control (Figure 9A,B). Utilizing peak sequences for each replicate, the DREME motif discovery tool distinguished the GGCTGGA logo as a common short conservative sequence bound by ZNF643 (Figure 9C). Most of the peaks were located in the vicinity of gene transcription start sites (TSSs) (Figure S10A,B), both in the genic and intergenic regions (Figure S10C,D). Within the genic region, ZNF643 preferentially resided within introns, while binding to coding sequences (CDS) was rare (Figure S10C,D). Notably, more ZNF643 peaks were found close to TSSs rather than transcription termination sites (TTSs) (Figure S10C,D). The annotation associated the peaks with protein-coding genes, pseudogenes, microRNAs, and long non-coding RNAs.

Next, we examined the biological significance of the genes related to the ZNF643 binding sites. We combined the peak overlapping genes and the genes with TSS closest to the ZNF643 binding loci. The Gene Ontology analysis detected various Biological Processes, including cell death, proliferation, adhesion, migration, developmental processes (e.g., angiogenesis, osteoblast differentiation), transcription, signal transduction (related to, e.g., EGFR, TGFβ), and other (Figure 9D). Under the cell adhesion category, we found such genes as tumor necrosis factor superfamily member 9 (*TNFSF9*), cadherins, collagen type IV alpha 6 chain (*COL4A6*), integrin subunit alpha V (*ITGAV*) and matrix metallopeptidase 2 (*MMP2*) (Supplementary File S3). Similarly to RNA-seq, we did not identify ferroptosis in our gene ontology analysis. Yet, several genes connected to ferroptosis were found among the genes associated with ZNF643 binding (e.g., elongation of very long chain fatty acids protein 5: *ELOVL5*, peroxisome proliferator-activated receptor gamma: *PPARG*, dipeptidase 1: *DPEP1*) (Supplementary Table S1). Interestingly, the Cellular Component and KEGG pathway analyses exhibited similar categories as in the case of the genes deregulated after ZNF643 silencing in the SKMES cell line (Figures S4B,C and S5A,B). Notably, KEGG analysis highlighted fluid shear stress and atherosclerosis, leukocyte transendothelial migration, AGE-RAGE signaling pathway in diabetic complications, and adhesion (Figure 9E). Apart from these categories, the ZNF643-related genes were shown to contribute to other signaling pathways (such as relaxin, prolactin, insulin signaling), immune system (e.g., T cell receptor signaling pathway, Yersinia infection), proteoglycans in cancer, and endocrine resistance (Figure 9E). In the Cellular Component analysis, the ZNF643-related genes were classified as parts of cell junction, synapse, and macromolecular complexes (Figure 9F). Interestingly, under the tab of Diseases, we identified a Tumor Suppressor category listing eight TSGs, including BCL10 immune signaling adaptor (*BCL10*), large tumor suppressor kinase 2 (*LATS2*), and polo-like kinase 2 (*PLK2*). The complete lists of the Biological Processes, Cellular Components, KEGG pathways, TSGs, and cell adhesion genes are provided in Supplementary File S3.

In the next step, we integrated ChIP-seq-annotated genes with the DEGs identified in our RNA-seq experiment on the SKMES shZNF643 cell line (Figure 6). We wondered whether ZNF643 might affect the expression of its targets. We identified two upregulated genes: Beta-1,4-N-acetyl-galactosaminyltransferase 3 (B4GALNT3) and TNFSF9, as well as two downregulated genes: Solute carrier organic anion transporter family member 1B3 (SLCO1B3), and spectrin repeat containing nuclear envelope family member 3 (SYNE3). The peak associated with *TNFSF9* was located at 5246 bp downstream of TSS (Figure S11A). The *SYNE3*-related peak was located 1931 bp upstream of TSS—it resided in the first intron and overlapped an ENCODE candidate cis-regulatory element (cCRE) (Figure S11B,C). For the validation analysis, we chose *TNFSF9* and *SYNE3*. We excluded other peaks because the *B4GALNT3* peak resided far from the TSS (39300 bp upstream), while the *SLCO1B3* peak was located closer to the TSS of the SLCO1B3-SLCO1B7 natural read-through transcript. In these circumstances, the interpretation of the results would be more difficult. For the validation, we also included a peak linked to the methionine adenosyltransferase 2 non-catalytic beta subunit gene (*MAT2B*), which was located 315 bp upstream of TSS in a cCRE and the first intron of the main isoform (or the first exon of some of MAT2B isoforms) (Figure S11D,E). In the single-gene ChIP-qPCR assay, all the selected target sequences showed enrichment in ZNF643-OE samples compared to WT (Figure 9G). We also investigated the expression of chosen target genes in the SKMES ZNF643-OE and shZNF643 cell lines using RT-qPCR assays (Figure 9H,I). Our analysis confirmed TNFSF9 upregulation and a trend of SYNE3 downregulation after ZNF643 silencing (Figure 9I). Interestingly, TNFSF9 mRNA level was also negatively correlated with ZN643 expression in TCGA LUSC samples (Figure 9J). We also observed a modest (<two-fold) upregulation of MAT2B in SKMES shZNF643 cells (Figure 9I), which implies that other genes may also be moderated by ZNF643. ZNF643 overexpression resulted only in increased SYNE3 levels, while TNSFS9 and MAT2B mRNAs remained unchanged, suggesting that the ectopic overexpression of ZNF643 does not evoke further silencing of those genes (Figure 9H).

## 3. Discussion

The exact role of ZN643/ZFP69B in human cells remains largely uncharacterized. To date, the most prominent function of ZNF643 is connected to ferroptosis, as it was upregulated in response to ferroptosis induction [8]. Recent data indicated that ZNF643, together with other ferroptosis-related genes (FRGs), may contribute to prognostic gene signatures in various cancers [10,11,12,13]. Moreover, in liver cancer, ZNF643 demonstrated oncogenic features in the in vitro experiments [10]. We found several FRGs among DEGs identified in our RNA-seq analysis after ZNF643 knock-down and within ZNF643-bound overlapped peaks and closest TSS in the ChIP-seq assay. These genes might be potential downstream ZNF643 targets implicated in ferroptosis signaling. However, additional in-depth investigations are needed to explore this possibility further. Our previous investigations revealed that the ZNF643 gene becomes upregulated in a number of various tumors compared to normal tissues, and its expression is associated with diverse clinical parameters, including patient survival [7]. Since those observations support the notion that ZNF643 impacts the tumorigenic phenotype, we wanted to explore this possibility in more detail.

Utilizing various public TCGA-based datasets, we confirmed and expanded the portfolio of tumors with augmented ZNF643 expression. Higher ZNF643 levels in TCGA datasets were also reported in liver cancer [10], triple-negative breast cancer [12], gastric cancer [28], and colon cancer [13]. However, the exact molecular mechanisms responsible for increased ZNF643 expression remain to be elucidated. The data suggested that in certain tumor samples, ZNF643 deregulation may be related to CNV events. Nevertheless, as some of the tumor types show a relatively high percentage of deletions and concomitant overall ZNF643 upregulation, other factors seem implicated in augmented ZNF643 transcription. Hypothetically, ZNF643 may be elevated due to the activities of specific non-coding RNAs from the tumor-associated mRNA–miRNA–lncRNA network, as such a co-expression module was described for gastric cancer [28]. Alternatively, aberrant signaling from cancer-related transcription factors may be involved in ZNF643 deregulation. For instance, we and others [10,11] observed that high ZNF643 expression occurs more frequently in tumors with mutated p53. Although we noticed significant changes in *ZNF643* promoter methylation status, we do not expect they may be causative factors driving altered ZNF643 expression. This is because the *ZNF643* promoter is hypomethylated (beta values < 0.1) in tumor and normal samples, so the modest changes in such a methylation profile are unlikely to affect expression.

Based on the GEPIA2 database, we found that high levels of ZNF643 correlate with poor prognosis in ACC, LIHC, SARC, and UVM, whereas an opposite association was observed in COAD, READ, and GBM. Our earlier analysis utilizing maximally ranked statistics showed that high ZNF643 levels contribute to shorter survival in BRCA and LUSC patients [7]. These outcomes corroborate other reports demonstrating similar prognostic potentials: A negative association with survival in liver cancer [10,11] and positive in colon cancer [13]. The most up-to-date literature on ZNF643 in cancer highlights its contribution to multi-gene prognostic models. ZNF643 was selected for those models due to its tumor-specific overexpression [28] and its linkage to ferroptosis and patients’ outcomes [10,11,12,13]. Importantly, the prognostic power of the models was tested on TCGA data and validated on external datasets available from GEO or ICGC. As a part of the described signatures, ZNF643 is also positively [10,11,12] or negatively [13] correlated with the parameters indicative of more advanced tumors. Altogether, these results imply that ZNF643 expression may be used as a component of a prognostic biomarker panel.

Furthermore, our analyses indicated that ZNF643 may play a role in cancer interactions with the immune system. First, we observed that in the majority of tumor types (apart from ACC, KICH, KIRC, MESO, PAAD, and THCA), ZNF643 expression correlates with an immunosuppressive phenotype. Moreover, high ZNF643 expression was frequently identified in the wound healing subtype, while lower expression was noticed in the inflammatory subtype defined by Thorsson and colleagues [14]. These differences could also be illustrated at the cellular level. In contrast to the inflammatory subtype, the wound healing subtype features decreased Th17 infiltration and a reduced content of Th1 compared to Th2 cells [14]. Such an association between ZNF643 expression and TILs (negative for Th1 and Th17 and positive for Th2) was also evident in the current study. The linkage with immunosuppressive features and immune-related processes was also documented in liver cancer, in which ZNF643 was investigated as a part of the prognostic FRG signature [10,11].

While the observations described above stem from the association studies, our RNA-seq and ChIP-seq data also underlined ZNF643’s involvement in immune signaling. ZNF643 silencing led to the deregulation of genes involved in various immune-related pathways, including infections, complement and coagulation cascade, and leukocyte transendothelial migration. Moreover, the genes associated with the ZNF643 binding site also pointed toward the immune system, i.e., response to TGFβ, infection, leukocyte transendothelial migration, and T cell receptor signaling pathway. After integrating ChIP-seq with RNA-seq and wet-lab validation of the sequencing data, we demonstrated that ZNF643 binds in the vicinity of the *TNFSF9* promoter, whereas ZNF643 silencing caused enhanced TNFSF9 expression. A significant negative correlation between ZNF643 and TNFSF9 expression was also observed in TCGA LUSC samples. These outcomes suggest that ZNF643 may directly repress TNFSF9, at least in LUSC. TNFSF9 is a cytokine that co-stimulates immune cells (e.g., T, natural killer) via interaction with its receptor, TNFRSF9 [29,30]. The agonists of TNFSF9/TNFRSF9 signaling show promising effects in anti-cancer immunotherapies [31,32]. It is involved in bidirectional cell-to-cell communication. TNFSF9-induced reverse signaling lowered proliferation, enhanced apoptosis, and cell cycle arrest in lung cancer cells [33]. In liver cancer, overexpression of TNFSF9 impaired proliferation, migration, invasion, and in vivo tumor growth and spread [34]. In situ, TNFSF9 appeared crucial for immunosurveillance since its expression was markedly reduced in the pre-invasive lesions that progressed to LUSC compared to regressive lesions [30]. It remains to be tested whether ZNF643 may be responsible for TNFSF9 inhibition during cancer progression.

Besides the influence on immune signaling, our in vitro and sequencing experiments revealed that ZNF643 is involved in cell migration, invasion, and adhesion. While TNFSF9 was previously demonstrated to play a role in these processes [34,35], *SYNE3*, another gene regulated by ZNF643, is implicated in migratory activities [36,37]. SYNE3 is a part of the linker of the nucleoskeleton and cytoskeleton (LINC) complex, which is crucial for the organization and dynamics of the perinuclear cytoskeleton [37,38]. SYNE3 silencing hindered the migratory abilities of lung cancer cells [36] and aortal cells subjected to shear stress, possibly due to the alterations within the perinuclear architecture [37]. ZNF643 seemed to positively regulate SYNE3 expression. Its knock-down led to reduced SYNE3 mRNA, while overexpression—to SYNE3 upregulation. Of note, ZNF643 silencing was also associated with lowered migratory and invasive properties of the SKMES cell line in our in vitro studies.

The reduced migratory potential may be partly explained by the modifications in cell adhesion [39] and the ontology occurring in our sequencing analyses. A number of genes associated with ZNF643 binding sites were related to adhesion, although it is not clear whether and how exactly ZNF643 may regulate their expression. Notably, the genes related to ZNF643 binding showed variable degrees of up- and downregulation in shZNF643 cells. It is likely that these repressing and activating effects may depend on the participation of ZNF643 in different protein complexes with contrasting influences on transcription. In addition, we observed that several genes involved in adhesion became deregulated after ZNF643 knock-down. For example, CNTN1, downregulated in shZNF643 cells, is a neural adhesion molecule linked to epithelial-to-mesenchymal transition (EMT), metastasis in lung cancer, drug resistance, and short survival [16,18,40,41]. Its high expression was connected to enhanced lung cancer cell migration and invasiveness [16]. Yan and colleagues showed that reduced CNTN1 expression in a lung cancer cell line decreases its invasion by E-cadherin downregulation via enhanced activation of AKT signaling [17]. We found that CNTN1 and AKT3 are downregulated in the shZNF643 SKMES cell line, and these cells migrate and invade at a lower rate. Other genes downregulated after ZNF643 silencing that exhibit oncogenic features are LGALS1 [19,20,21,22] and EDIL3 [23,24,42]. In cervical and NSCLC cell lines, LGALS1 positively correlated with proliferation, migration, and invasion [22], while in oral and ovarian cancer—with advanced tumor and lymph node metastasis [20,21]. EDIL3 was shown to be crucial for EMT regulation in various tumors [23,24,42]. It enhanced cell growth, mobility, and invasiveness [23,24], while in pancreatic cancers, it was associated with TNM stage and shorted survival [42]. Furthermore, we noticed that ZNF643 silencing led to the upregulated expression of the tight junction gene, CLDN11. CLDN11 functions as a tumor suppressor, and its overexpression reduces cell migration and invasiveness [25,26]. While altered migration and invasion abilities of the cell may be related to ZNF643-mediated differences in the expression of the abovementioned genes, the molecular mechanisms implicated in this transcriptional deregulation are yet to be elucidated.

## 4. Materials and Methods

### 4.1. Databases Used for the Analysis of Pan-Cancer Omics Data Related to ZNF643

The UALCAN database was used to examine the differential expression and promoter methylation of ZNF643 across multiple tumors compared to normal specimens. Moreover, it was used to assess ZNF643 expression in relation to *TP53* mutation status (https://ualcan.path.uab.edu/, accessed on 12 July 2023) [43,44]. We used the TISIDB database (http://cis.hku.hk/TISIDB/index.php, accessed on 5 July 2023) [45] to investigate the differences in ZNF643 expression within various molecular and immune subtypes, tumor stages, and grades. We also analyzed the connection between ZNF643 expression and distinct features characteristic of tumor-immune system interactions (i.e., tumor-infiltrating lymphocyte (TIL) signature, the expression of major histocompatibility complex (MHC) molecules, immunoinhibitors, immunostimulators, chemokines, and chemokine receptors). GEPIA2 portal (http://gepia2.cancer-pku.cn/#index, accessed on 5 July 2023) [46] provided data on the prognostic potential of the ZNF643 gene expression level. To perform the log-rank test and hazard ratio estimation, patients were divided into high- and low-expression groups based on the median ZNF643 expression level. Furthermore, we probed cbioportal (https://www.cbioportal.org/, accessed on 12 July 2023) [47,48] and GSCA (http://bioinfo.life.hust.edu.cn/GSCA/, accessed on 12 July 2023) [49,50] to evaluate the mutation status of *ZNF643*. The data highlighting the correlation between copy number variation (CNV) and ZNF643 expression were downloaded from GSCA. The molecular characterization of SKMES and H2073 cell lines was performed with the cell line Data Explorer tool from depmap portal. CNV status for *ZNF643* was retrieved from https://depmap.org/portal/gene/ZFP69B?tab=characterization&characterization=copy_number_absolute (accessed on 13 October 2023). The information on *TP53* status in those cell lines was available from https://depmap.org/portal/gene/TP53?tab=characterization&characterization=mutation (accessed on 25 October 2023).

### 4.2. ZNF643 Gene Expression Knock-Down and Overexpression in a Cell-Line Model

For the in vitro studies, we used American Type Culture Collection (ATCC, Manassas, VA, USA) lung cancer cell lines: H2073 (#CRL-5918) for lung adenocarcinoma and SKMES (#HTB-58) for lung squamous cell carcinoma that were obtained from Dr. Triantafillos Liloglou from Edge Hill University, Medical School (Liverpool, UK). Lentiviral particles were prepared using HEK-293T (#CRL-3216, ATCC). Lung cancer cell lines were cultured in DMEM-F12 (Biowest, Nuaillé, France), supplemented with 10% FBS (EURx, Gdańsk, Poland) and 1% penicillin/streptomycin (Biowest, Nuaillé, France). Cells were cultured in a humidified incubator (37 °C, 5% CO_2_). Trypsin (Biowest, Nuaillé, France) was used to detach the cells during passaging.

We used a mix of three shRNA sequences targeting ZNF643 mRNA to obtain the most efficient knock-down (5′-GCCTTACAGAATGATATTTCG-3′; 5′-GGCCTAATGATGGAAAGATTT-3′, 5′-GCAGCATGTATTCCACCTTGG-3′). The shRNA sequence complementary to luciferase (5′-CAGCGATGACGAAATTCTTAG-3′) was used as a control. The shRNA sequences were cloned into a pLV-THEM-GP transfer vector constructed in our laboratory by adding an EcoRI restriction site to the pLVTHM plasmid (Addgene plasmid #12247) [51]. Lentiviral particles were produced employing the 2nd generation packaging system with psPAX2 and pMD2.G plasmids in HEK-293T cells. To obtain ZNF643 overexpression (ZNF643-OE) in the SKMES cell line, we used an HA-tagged *ZNF643* coding sequence delivered with a pEXPpSIN-TRE-GW-ZNF643-3xHA lentiviral plasmid, kindly gifted by Prof. Didier Trono from the École Polytechnique Fédérale de Lausanne (Lausanne, Switzerland) [52]. ZNF643 overexpression (ZNF643-OE) was induced with 2 µg/mL doxycycline (Sigma-Aldrich, St. Louis, MO, USA).

### 4.3. RNA-seq and Differential Gene Expression Analysis

Quick-RNA Miniprep Kit (Zymo Research, Irvine, CA, USA) was used to purify total RNA from H2073 and SKMES cell lines according to the producer’s instructions. We used the 6000 Nano Assay on a Bioanalyzer (Agilent Technologies, Waldbronn, Germany) to estimate the quality and quantity of prepared samples. Next, the samples were outsourced to Macrogen Europe for pair-end RNA sequencing on a NovaSeq 6000 Sequencing System (Illumina, San Diego, CA, USA). HISAT2 was used to map the trimmed reads to the UCSC hg38 human reference genome, and known genes and transcripts were assembled with StringTie. The abundance of gene/transcript was calculated in the read count and represented as a normalized value (FPKM—fragments per kilobase per million or TPM– transcript per kilobase per million mapped reads). We performed differentially expressed genes (DEGs) using edgeR. Significant expression differences were considered when genes met the following criteria: |fc| >= 2 and exactTest raw *p*-value < 0.05. We calculated the fold change and performed the normalization with DESeq2 (v. 1.28.0). The RNA-seq analysis from WT, shLUC, and shZNF643 was performed in biological triplicates.

We used the Database for Annotation, Visualization and Integrated Discovery (DAVID) [53,54] to perform Biological Processes, Cellular Components, and KEGG pathway analysis for DEGs identified in RNAseq. We combined identified DEGs for shZNF643 obtained from two different comparisons (shZNF643 vs. WT and shZNF643 vs. shLUC) separately for H2073 and SKMES cell lines. Only genes present in both comparisons showing simultaneous overexpression or downregulation were chosen for further analysis. The list was narrowed to 108 DEGs for H2073 and 370 for the SKMES cell line. Identified Biological Processes, Cellular Components, and KEGG pathways were considered significant at *p* < 0.05 and FDR < 0.25. The RNA-seq data are available from Gene Expression Omnibus (GEO): GSE242003.

### 4.4. Western Blot

The protein fraction from the SKMES ZNF643-OE cell line was isolated using RIPA buffer with protease inhibitors (Sigma-Aldrich, St. Louis, MO, USA). Cells were incubated for 30 min on ice, centrifuged (15,000 rpm/4 °C/15 min), and the supernatant was collected. We used the Pierce™ BCA Protein Assay Kit (Thermo Fisher Scientific, Waltham, MA, USA) to assess protein concentration. Next, 4× Laemmli Sample Buffer (BioRad, Hercules, CA, USA) was added to 50 μg of the isolated protein, and the samples were mixed and denatured (98 °C/5 min). We conducted electrophoresis using Tris/Glycine/SDS buffer (BioRad, Hercules, CA, USA), Mini-PROTEAN TGX Precast Gel (BioRad, Hercules, CA, USA), and Precision Plus Protein Kaleidoscope Prestained Protein Standards (Bio-Rad, Hercules, CA, USA). Afterward, the proteins were transferred on a PVDF membrane with a Trans-Blot Turbo Transfer Pack (BioRad, Hercules, CA, USA). Membrane blocking was performed with 5% milk in TBST buffer for 30 min at room temperature (RT). The membrane was further incubated with mouse anti-HA antibody (#32-6700 Invitrogen, Waltham, MA, USA) in a blocking solution (4 °C/overnight). After double washing with 0.1% TBST buffer for 15 min, the membrane was incubated with a secondary rabbit anti-mouse HRP-conjugated antibody (ab6728 Abcam, UK) (1 h/RT). We used WesternBright Quantum solutions (Advansta, San Jose, CA, USA) and a G-Box gel documentation device (Syngene, Cambridge, UK) for the visualization. For loading control, we performed an incubation with anti-GAPDH primary antibody (Ab) (ab9485 Abcam, Cambridge, UK) followed by secondary goat anti-rabbit Ab (ab6721 Abcam, Cambridge, UK).

### 4.5. Immunofluorescence Cell Staining

WT and ZNF643-OE variants of SKMES cell lines were treated with 2 µg/mL doxycycline for 48 h to induce ZNF643 expression. The samples were further fixed with 4% paraformaldehyde (Agar Scientific Ltd., Rotherham, UK), permeabilized with ice-cold methanol, and blocked with 1% BSA in PBS for 15 min. Next, 1:100 anti-HA antibody (Invitrogen, USA) and 1:100 DyLight 650-labeled beta-actin monoclonal antibody (Thermo Fisher, USA) were added for the overnight incubation at 4 °C. Afterwards, the cells were washed with 1% BSA in PBS and probed with Alexa Fluor^®^488-conjugated anti-rabbit secondary antibody (Jackson Immuno Research, West Grove, PA, USA) at a dilution of 1:200 (1 h/RT). Finally, the cells were washed in PBS and immersed in a Fluoroshield mounting medium containing DAPI (Sigma-Aldrich, USA) to visualize cell nuclei. The Olympus FV1000 scanning confocal microscope (Shinjuku, Shinjuku, Japan) with a laser diode (405 nm) and argon laser was utilized for the image acquisition. The specimens were visualized with a 60× objective, a 1.4 NA oil immersion lens, and FLUOVIEW Viewer software (ver. 4.1).

### 4.6. Assessment of Cell Proliferation

To evaluate the effect of knock-down or overexpression of ZNF643 on cancer cells, we applied an MTT colorimetric and real-time confluency-based assays (IncuCyte^®^, Sartorius, Edgewood, NY, USA). For the MTT assay, we seeded the cells on a 96-well culture plate in an optimized amount: 2000 cells (SKMES) and 7500 cells (H2073). For the real-time confluency-based assay, the cells were seeded at a density of 5000 cells (SKMES) and 15,000 cells (H2073). For the real-time experiments, we cultured the cells inside an IncuCyte^®^ instrument (Sartorius, Göttingen, Germany) in a humidified CO_2_ incubator, and the pictures were taken every 3 h to illustrate the level of cell confluency. We used the IncuCyte^®^ software (Incucyte-2021B software, Göttingen, Germany) to compare the confluency level at different time points and to calculate the cell proliferation rate. To normalize the results, we chose a starting time-point when the control cells achieved around 20% confluence on a culture plate.

### 4.7. Analysis of Cell Cycle and Apoptosis

Cell cycle and apoptosis in the cells with ZNF643 knock-down were evaluated with flow cytometry-based assays on the FACSAria cytometer (BD Sciences, Franklin Lakes, NJ, USA). For cell cycle analysis, we stained the cells with propidium iodide (PI) (Sigma-Aldrich, USA). The cells were collected, washed with DPBS (Biowest, France), and fixed with ice-cold 70% ethanol (POCH, Gliwice, Poland) for 30 min on ice. After that, the cells were incubated with 5 μg RNAse (EurX, Poland) and stained with 200 μL of 50 μg/mL PI (30 min/37 °C). We conducted a double staining protocol with PI and Annexin V-APC (ThermoFisher, USA) to analyze apoptosis. Briefly, after harvesting, the cells were washed in 1mL of μL of Annexin V Binding Buffer, centrifuged and resuspended in 100 μL of Annexin V Binding Buffer (ThermoFisher, USA) and 5 μL of AnnV-APC (for 10 min in the dark). Then, we washed the cells in 500 μL of Annexin V Binding Buffer, centrifuged them, and resuspended them in 200 μL of Annexin V Binding Buffer with 5 μL PI. The downstream analysis allowed us to divide the cells into four groups: PI−/AnnV− viable cells, PI+/AnnV− necrotic cells, PI+/AnnV+ late apoptosis, and PI−/AnnV+ early apoptosis.

### 4.8. Migration and Invasion

To determine the potential changes in migration and invasion of cancer cells with altered ZNF643 expression, we performed relevant real-time assays using the IncuCyte^®^ Instrument (Sartorius, Göttingen, Germany). The procedures were performed according to the manufacturer’s recommendations. We seeded 6 × 10^4^ SKMES cells per well on a 96-well culture plate pre-coated with 100 µg/mL Matrigel™ (Invitrogen, USA) to obtain 100% confluency the following day. Then, we added 3% mitomycin (3 h/37 °C) to prevent further proliferation. To create similar scratches across all wells, we used the WoundMaker device (Sartorius, USA). The cellular debris was removed by double washing with PBS. To examine cell invasion, the plate was chilled on ice for 5 min, and the cells were covered with 50 μL of 2 mg/mL Matrigel™. We incubated the cells for two days in the IncuCyte Instrument (Sartorius, Göttingen, Germany), and the images documenting wound confluence were taken every six hours. Wound coverage at different time-points was assessed with the IncuCyte^®^ software (Incucyte-2021B software, Göttingen, Germany). During optimization, the H2073 cell line showed negligible migratory and invasive properties, hindering the interpretation of the results. Therefore, it was not included in these tests.

### 4.9. Chromatin Immunoprecipitation Coupled with Next-Generation Sequencing (ChIP-seq) Analysis

Chromatin immunoprecipitation (ChIP) was carried out with the HighCell# ChIP Kit (Diagenode, Seraing, Belgium) according to the producer’s protocol; however, minor modifications were introduced. Briefly, after induction of ZNF643 overexpression in the SKMES cell line, the cells were harvested and counted. We used 10 mln cells for crosslinking with 1% methanol-free formaldehyde (Invitrogen, UA) for 8 min in RT. The fixation was stopped with glycine, and after extraction of cell nuclei, the samples were subjected to fragmentation with the Bioruptor sonicator (Diagenode, Belgium) by applying four runs consisting of ten cycles (30 s on/30 s off), followed by short vortexing after each cycle. After sonication, the lysates were spun and purified on 50 µL pre-washed Pierce™ Protein A Agarose beads (Invitrogen, USA) for 2.5 h at 4 °C. After purification, the lysates were centrifuged and transferred onto the magnetic beads covered previously with 2 µg rabbit anti-HA ChIP-grade antibody (#ab9110, Abcam, USA). The immunoprecipitation was performed at 4 °C overnight with gentle rotation (40 rpm). For DNA elution and decrosslinking, we utilized the iPURE kit (Diagenode, Belgium) according to the producer’s instructions. DNA was quantified with the QuantiFluor dsDNA test (Promega, Madison, WI, USA).

Further quality control, next-generation sequencing on the NovaSeq Illumina Platform, and downstream bioinformatics analyses were performed by an outsourced company, Novogene (UK). The immunoprecipitated and input DNA samples were sequenced in biological duplicates in a paired-end (PE-150) mode. Read quality control was performed with the FastQC software (https://www.bioinformatics.babraham.ac.uk/projects/fastqc/, accessed on 30 March 2021). After trimming the adapter sequences and poor-quality bases, the reads were aligned to the UCSC hg38 human reference genome with the BWA tool [55]. MAPQ was used to assess mapping quality. The peaks were called with MACS2 software (v. 2.1.0) [56] and annotated with PeakAnnotator [57]. The DREME tool was applied to search for significant motif sequences related to the peaks [58]. Differential enrichment analysis between WT and ZNF643-OE cell variants was performed with diffbind software https://www.bioconductor.org/packages//2.10/bioc/html/DiffBind.html, accessed on 30 March 2021). Gene Ontology and KEGG pathway analysis of the genes related to the enriched (>2-fold) peaks (including overlapping genes and the genes with the closest TSS to the peak) were carried out with the DAVID database (accessed on 8 August 2023) [53,54]. For the data presentation, we chose the Biological Processes (BP ALL), Cellular Component (CC1), and KEGG pathways with non-adjusted *p* < 0.05. The hg38 genome from the Genome Browser (accessed on 13 August 2023) [59] was used to visualize ZNF643-bound sequences. We included additional tracks, namely, CpG Islands and ENCODE Candidate Cis-Regulatory Elements (cCREs) combined from all cell types, to gain information about the functional regions related to the ZNF643 binding sites. The ChIP-seq data are deposited in the GEO repository under the accession number (GSE242003).

### 4.10. RT-qPCR and ChIP-qPCR

We extracted RNA from the cell line variants (WT, shLUC, and shZNF643) in 3 biological replicates with the GeneMATRIX Universal RNA Purification Kit (EurX, Poland) according to the manufacturer’s instructions. Next, we prepared cDNA from 1ug RNA using the smART First Strand cDNA Synthesis Kit (EurX, Poland). We used LightCycler 480 SYBR Green Master Mix (Roche, Basel, Switzerland) or iTaq™ Universal SYBR^®^ Green Supermix (BioRad, USA) to conduct qPCR on a LightCycler 480 instrument (Roche, Switzerland). The comparative Ct method (RQ = 2^−ΔΔCt^) was utilized to calculate the relative quantification values. For the RT-qPCR analysis, we used the following primers (5 μM primer FW + RV mix per reaction): ESD: FW 5′-TGATCAAGGGAAAGATGACCA-3′, RV 5′-AACCCTCTTGCAATCGAAAA-3′; ZNF643: FW 5′-GTGGGAGGATGTGACTAAGATGT-3′, RV5′-ACTTTCGCCCTGGGTCTC-3′; TNFSF9: FW 5′-GAGCTTTCGCCCGACGAT-3′, RV 5′-GGGTCACTGTACCAGCTCAG-3′; SYNE3: FW 5′-GCTGAGTACGATGCAGTGAA-3′, RV 5′-GTTGGAACTCGTCCACACC-3′; MAT2B: FW 5′-AGCTCTCTATACACTTTGTTCCC-3′, RV 5′-ATTCTTTGTGTACAGCTCTGCC3′. CNTN1: FW 5′-CTGCTGTCAAGTAGCCAGGG-3′, RV: 5′-TCTTCTATACCATGTAAACTCTGCT-3′; EDIL3: FW 5′-CCCAGTTCGGCAAAGGTGAT-3′, RV 5′-GGGACCTGCTGAAGTTGGTT-3′; CLDN11: FW 5′-GTGACCACCTCCACCAATGA-3′, RV 5′-GCACGTAGCCCGGCAG-3′; LGASL1: FW 5′-AATCATGGCTTGTGGTCTGG-3′, RV 5′-CGAAGCTCTTAGCGTCAGGA-3′. ESD was used as an internal control in lung cancer cell lines. For the ChIP-qPCR analysis, we used the primers listed below: *TNFSF9*: FW 5′-TCCTTGGAGAGCGACCTTCT3′, RV 5′-AGACACACACATGCCCTCAG-3′; *SYNE3*: FW 5′-TGGAGCTAAGGCCGGTGTAA-3′, RV 5′-CACACCTCGCAAGCTCACAA-3′; *MAT2B*: FW 5′-GCTTTGCAGGCGTATTCCG-3′, RV 5′-CCGTGTGCGCTGAGATAAAC-3′, *ACTB*: FW 5′-TTTGCACTTTCTGCATGTCC-3′, RV 5′-GGGGTGTTGAAGGTCTCAAA-3′. *ACTB* served as a non-specific region, constituting an internal control.

## 5. Conclusions

In the current study, we demonstrated that ZNF643 is upregulated in most tumor types available in TCGA datasets, and its deregulation may be partially linked to CNV events. Its expression was related to various molecular and immune subtypes and clinicopathological parameters, including tumor stage, grade, and survival. The RNA-seq and ChIP-seq analyses detected genes linked to several cancer-related processes, such as growth, adhesion, and immune system. Moreover, our data indicate that ZNF643 may positively correlate with migration and invasion in a cell-type-dependent manner. Altogether, our data demonstrated that ZNF643 may influence immune signaling (via TNFSF9) and cell adhesion (via CLDN11, CNTN1, EDIL3, LGALS1), and as such, it may confer pro-tumorigenic features. Further in vivo experiments are warranted to explore ZNF643 function in cancer.

## Data Availability

The transcriptomic RNA-seq and ChIP-seq data are available in the GEO under the accession number GSE242003.

## Supplementary Materials

The following supporting information can be downloaded at: https://www.mdpi.com/article/10.3390/ijms242216380/s1.
