# Supplementary material for: ZNF643/ZFP69B Exerts Oncogenic Properties and Associates with Cell Adhesion and Immune Processes
Source: Int J Mol Sci. 2023 Nov 15;24(22):16380. doi: 10.3390/ijms242216380 (PMC10671213; doi:10.3390/ijms242216380)
Supplement: Supplementary file 1 [file ijms-24-16380-s001.zip › Supplemental Figures.pdf]

Supplemental Figure 1

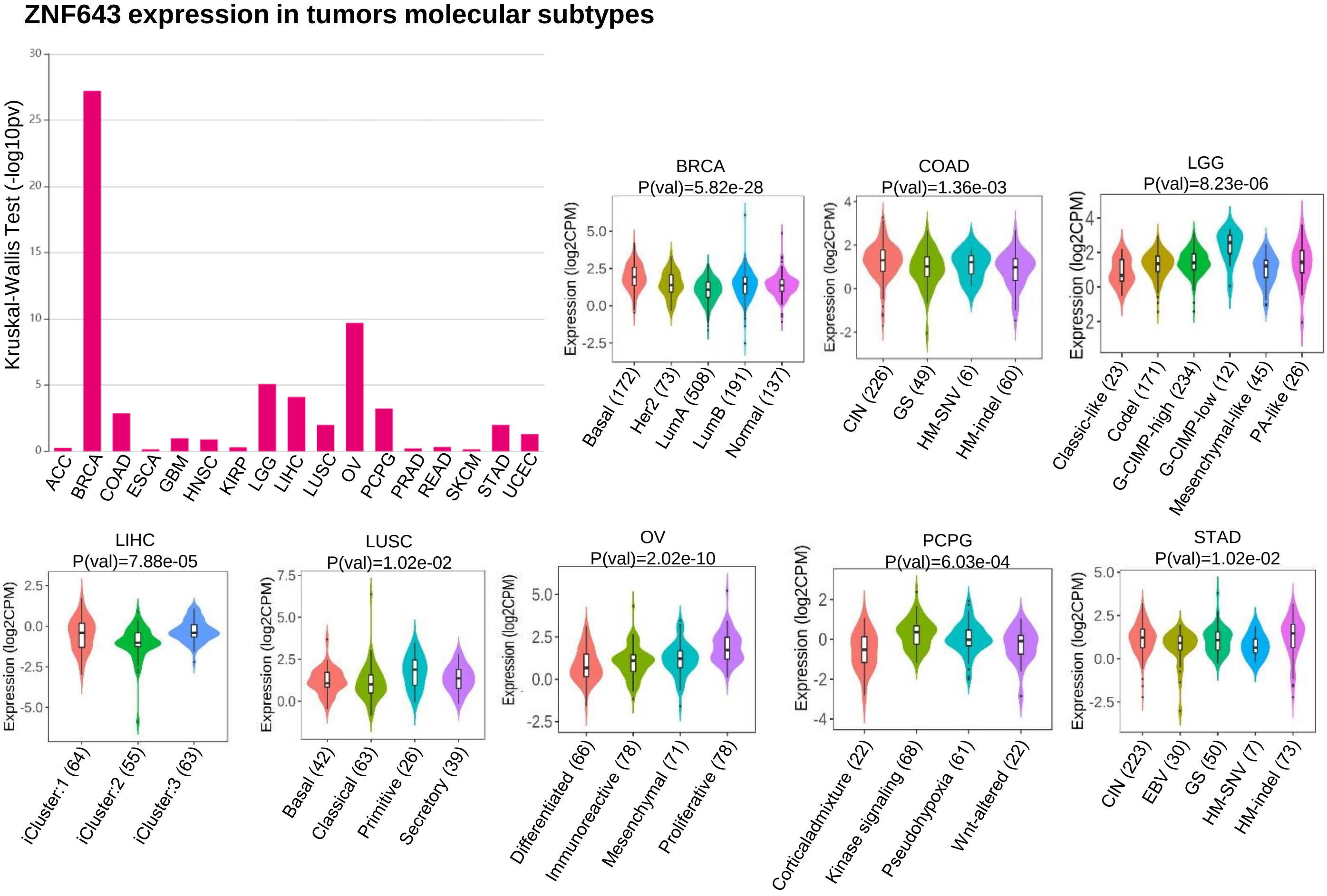

Figure S1. The expression signature of ZNF643 in tumor molecular subtypes. ZNF643 expression was significantly associated with a molecular subtype in eight analyzed tumor cohorts demonstrated on the tumor type-related graphs. The number of samples in each subtype is presented in brackets. The p-value was calculated using the Kruskal-Wallis test.

Supplemental Figure 2

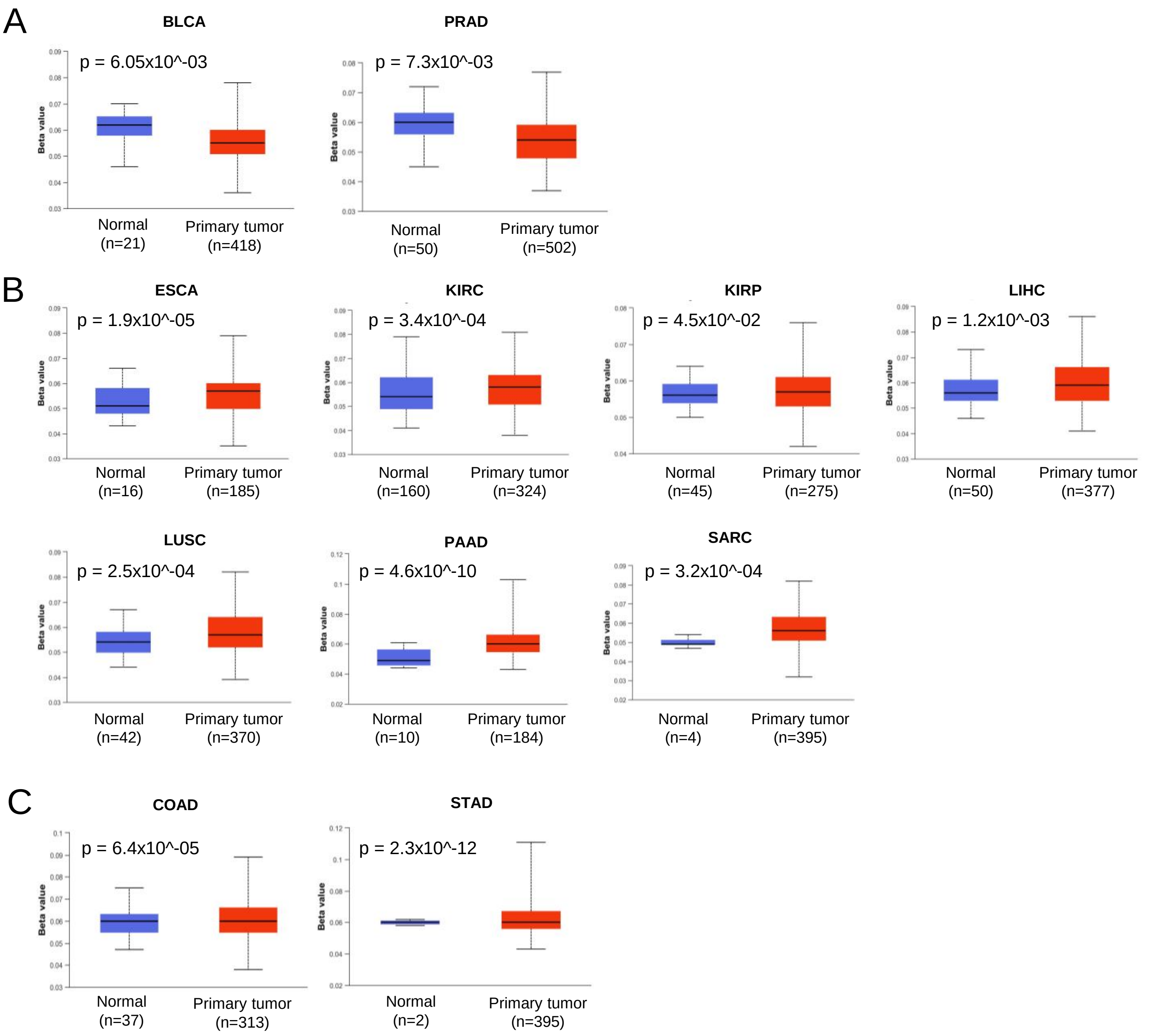

Figure S2. ZNF643 promoter methylation. Boxplots represent ZNF643 promoter methylation in normal (blue) and tumor (red) samples in distinct TCGA tumors based on the UALCAN database. Student t-test was used to assess the significance of a difference between normal and tumor samples. The boxplots represent (A) hypomethylation in tumor samples, (B) hypermethylation in tumor samples, (C) no difference in median methylation between normal and tumor samples.

Supplemental Figure 3

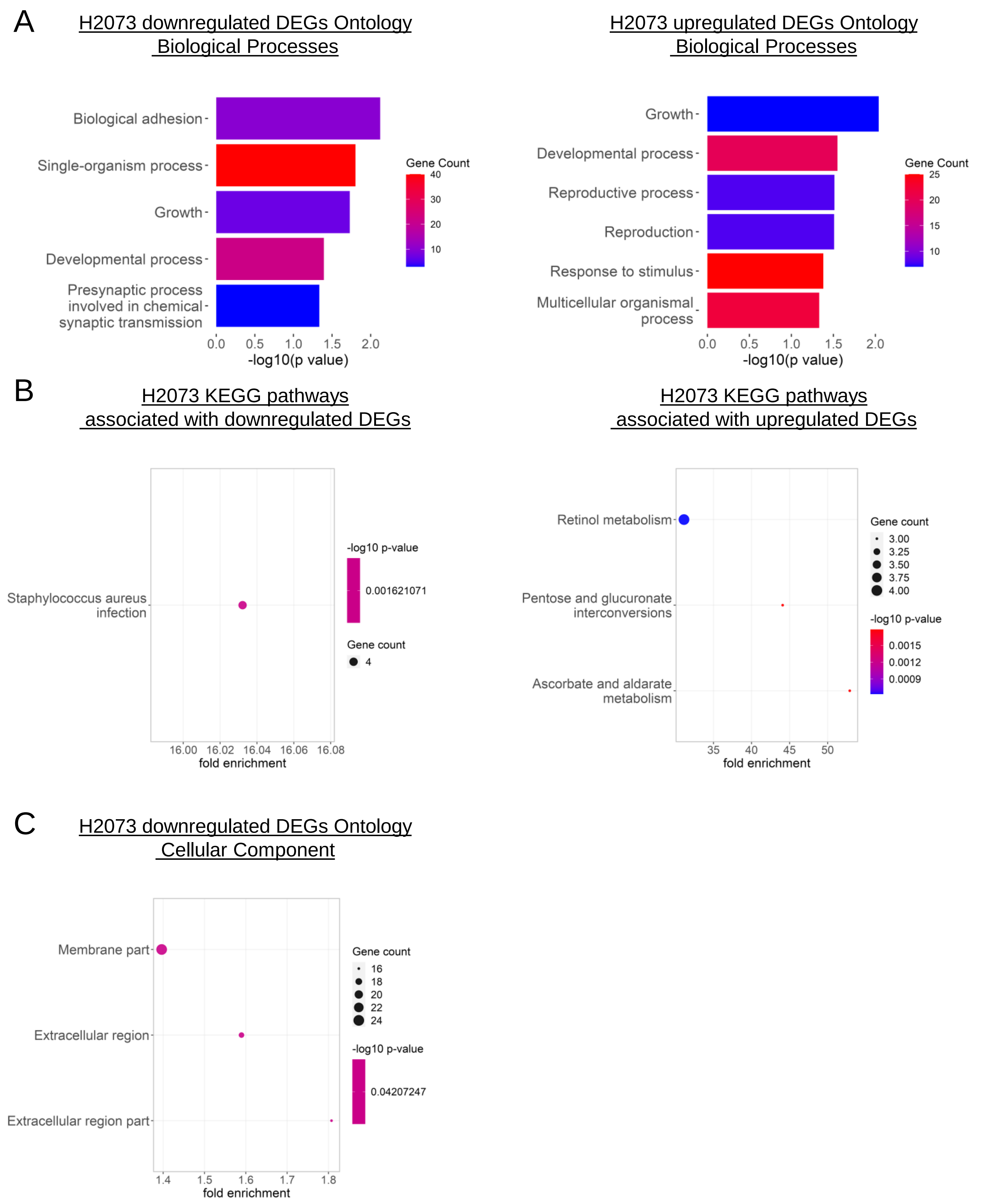

Figure S3. GO analysis of DEGs in H2073 shZNF643 cell line. (A) Biological Processes (BP1), (B) KEGG enriched pathways, and (C) Cellular Components associated with down- and up-regulated DEGs in H2073 cell line. The color scale on barplots represents the number of DEGs involved in each process. Bubble plot: X-axis – fold enrichment; color scale – p-value; bubble size – the number of genes involved in each cellular component

.

Supplemental Figure 4

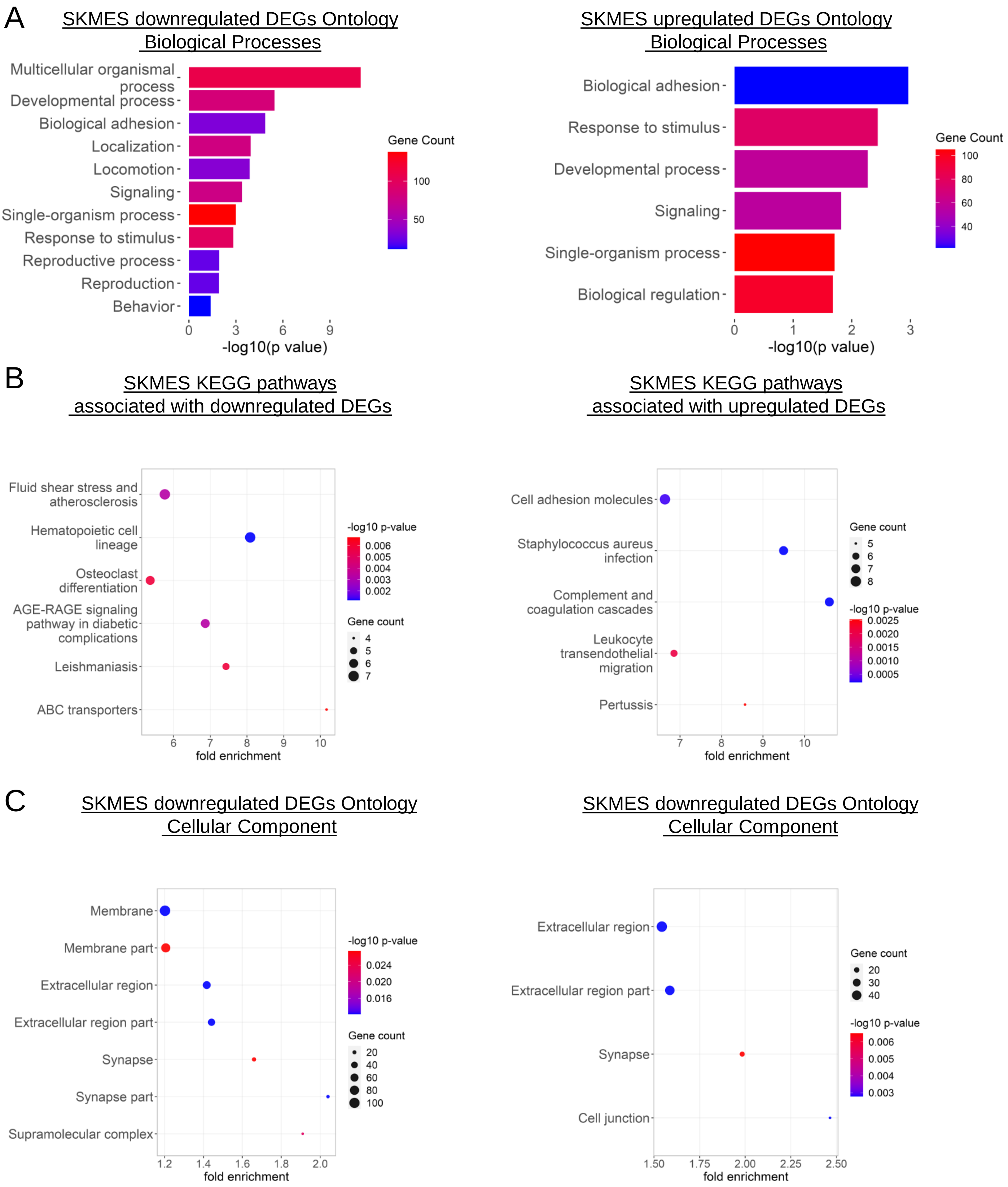

Figure S4. GO analysis of DEGs in SKMES shZNF643 cell line. (A) Biological Processes (BP1), (B) KEGG enriched pathways, and (C) Cellular Components associated with down- and up-regulated DEGs in SKMES cell line. The color scale on barplots represents the number of DEGs involved in each process. Bubble plot: X-axis – fold enrichment; color scale – p-value; bubble size – the number of genes involved in each cellular component

.

Supplemental Figure 5

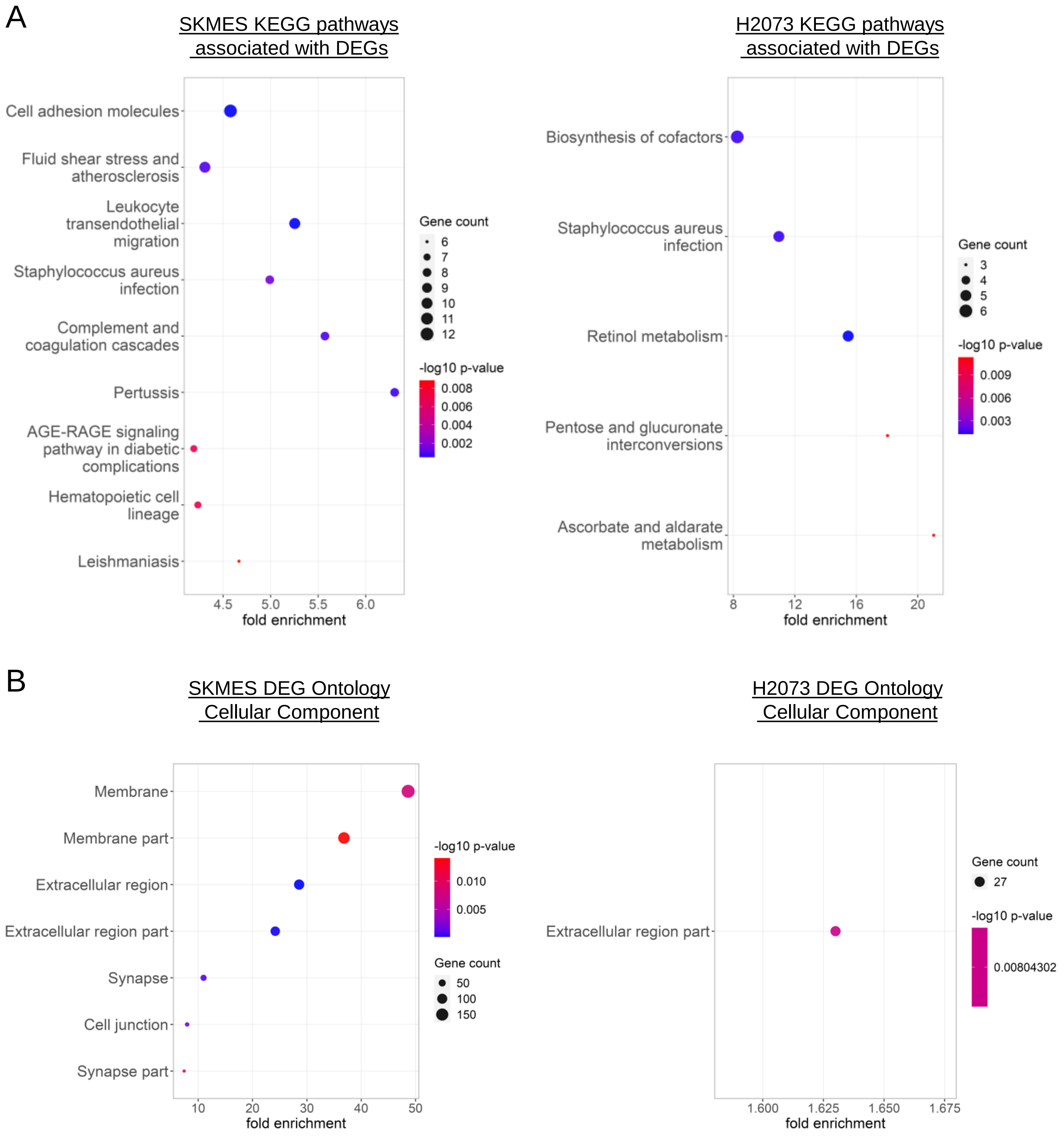

Figure S5. GO analysis of DEGs in lung cancer shZNF643 cell lines. (A) KEGG enriched pathways, and (B) Cellular Components associated with all (upregulated and downregulated) DEGs in SKMES and H2073 cell lines. X-axis – fold enrichment; color scale – p-value; bubble size – the number of genes involved in each cellular component

.

Supplemental Figure 6

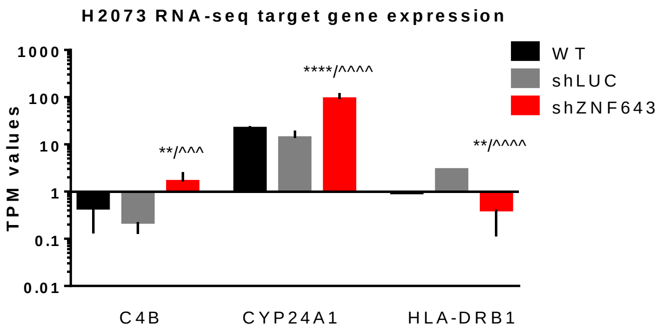

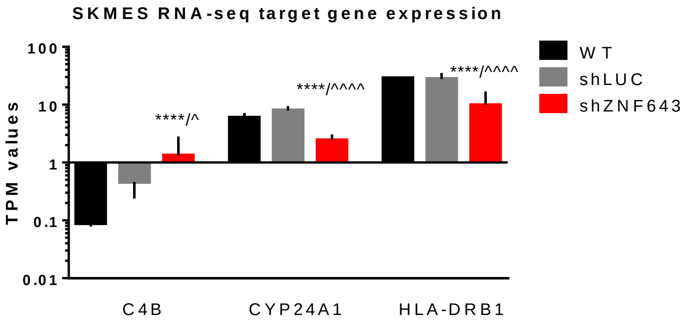

Figure S6. Normalized expression for ZNF643 target genes based on RNA-seq analysis. The graphs represent TPM values of the genes that are deregulated in H2073 (left) and SKMES (right). Complement C4B Chido blood group (C4B), Cytochrome P450 Family 24 Subfamily A Member 1 (CYP24A1), Major Histocompatibility Complex, Class II, DR beta 1 (HLA-DRB1). P-values: * vs. WT; ^ vs. shLUC; ^ p < 0.05; ** p < 0.01; ^^^ p < 0.001, ****/^^^^ p < 0.0001.

Supplemental Figure 7

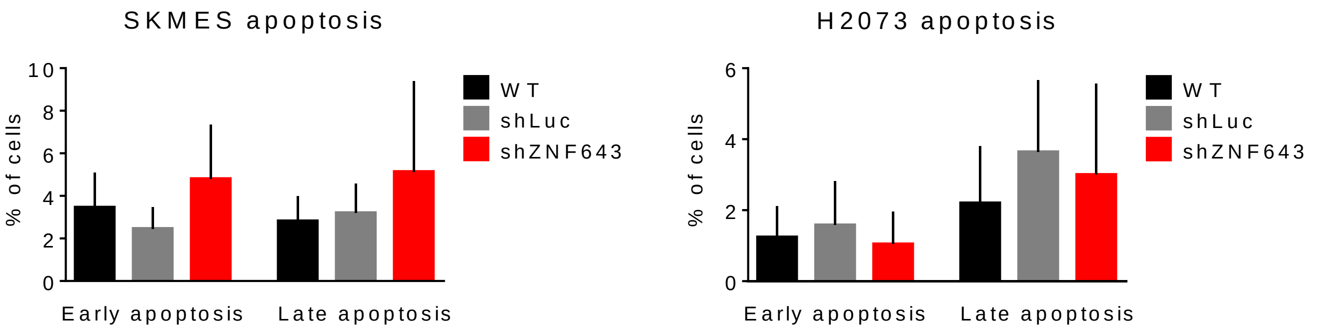

Figure S7. Analysis of apoptosis in shZNF643 lung cancer cell lines. The study of apoptosis was performed by flow cytometry. The bar graphs represent statistics for at least three biological replicates.

Supplemental Figure 8

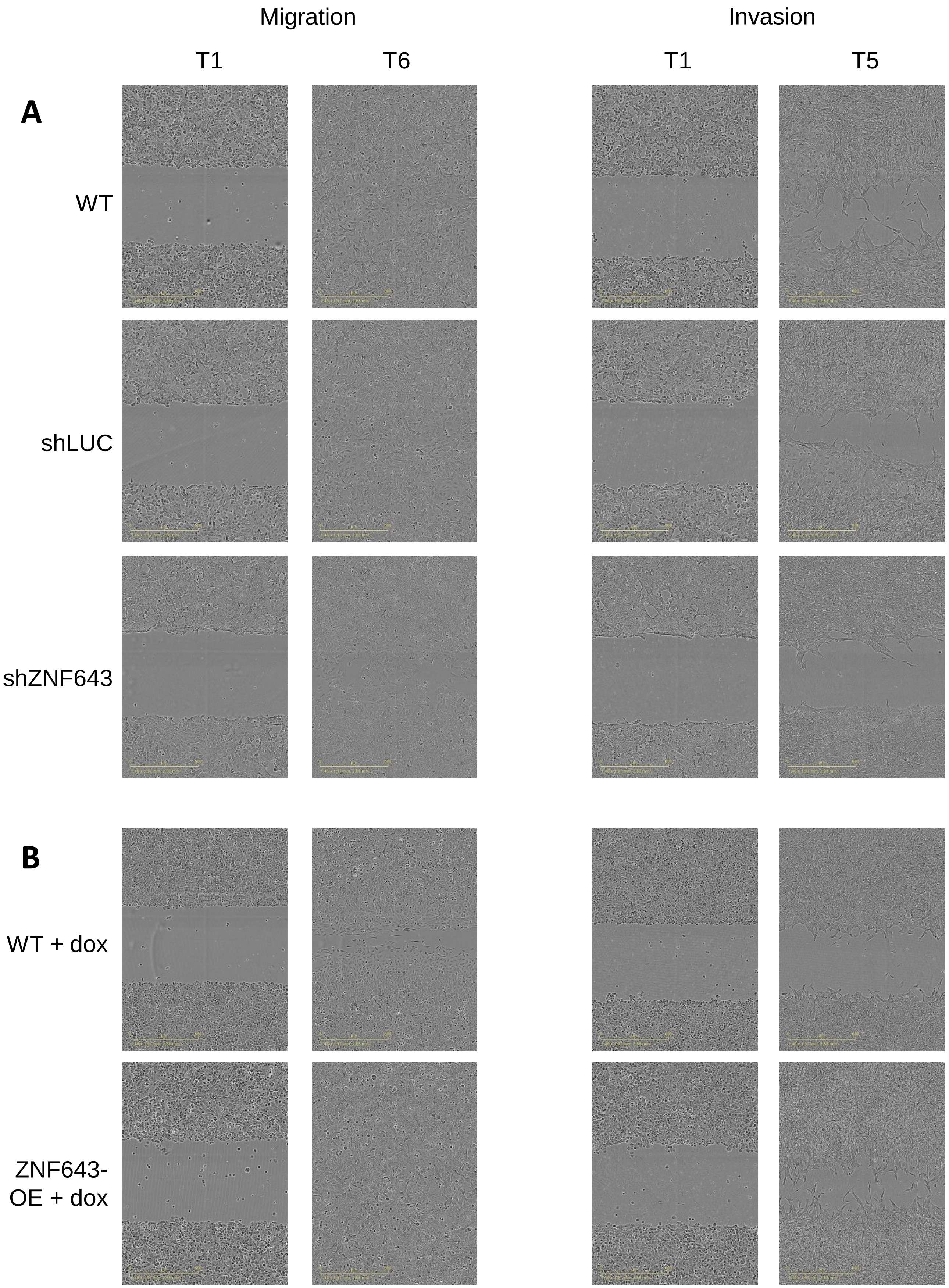

Figure S8. Migration and invasion in lung cancer cell lines. The images represent the following SKMES cell variants: shZNF643 (A) and ZNF643-OE (B). The images were acquired with the Incucyte device (Sartorius). The scale in the lower left corner represents 400µm.

Supplemental Figure 9

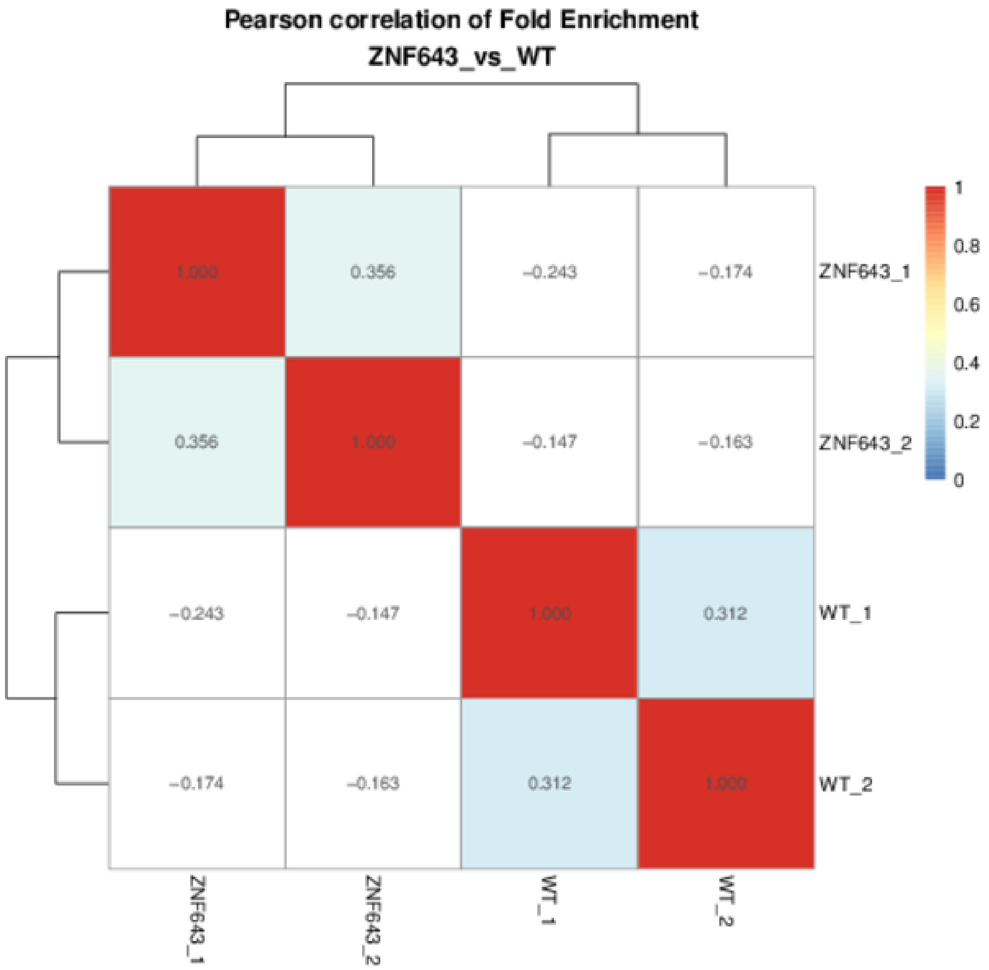

Figure S9. Pearson correlation of Fold Enrichment values in each replicate of ZNF643-OE and WT SKMES cell lines. The Fold enrichment was calculated as a ratio of a number of reads enriched in peak area per milion reads (RPM) in ChIP to RPM in Input. The positive correlation values are marked in colors as shown in the scale on the right.

Supplemental Figure 10

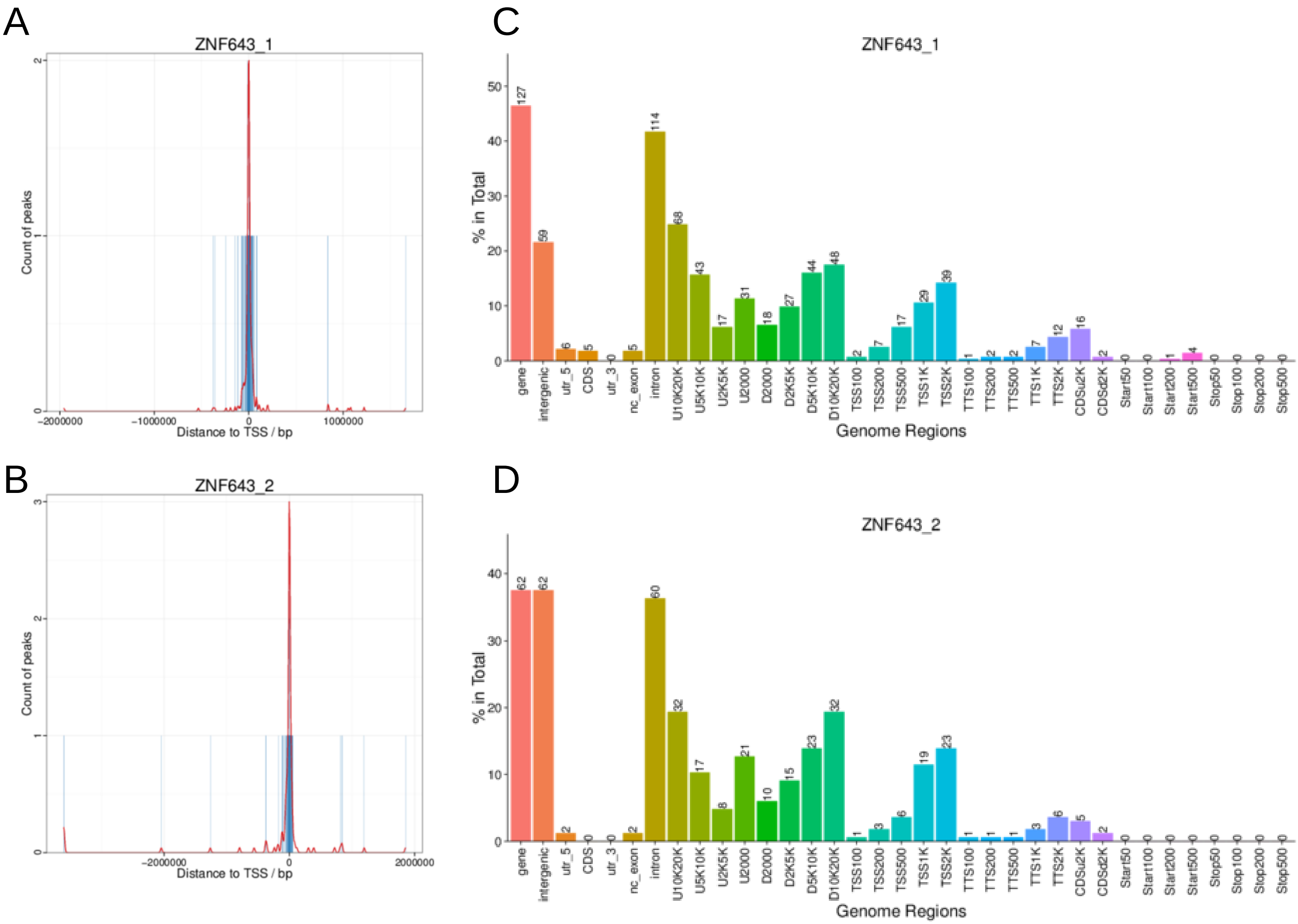

Figure S10. ZNF643 peak distribution within the genomic region. (A, B) Distribution of the values depicting the distance between the peak and related TSS for both replicates. (C, D) ZNF643 peak distribution among various functional genomic regions listed on the Y axis. The X axis represents the ratio of the peaks within the functional area to the total number of peaks. The number of peaks within the functional regions is provided on each bar's top. U2000 means 2000 bp upstream (D2000 – downstream), CDSu2K and SDCd2K denote upstream and downstream 2 Kb of CDS. TSS200, top 100, start 100 means 100bp centered with TSS, TTS, start-codon, and stop-codon.

Supplemental Figure 11

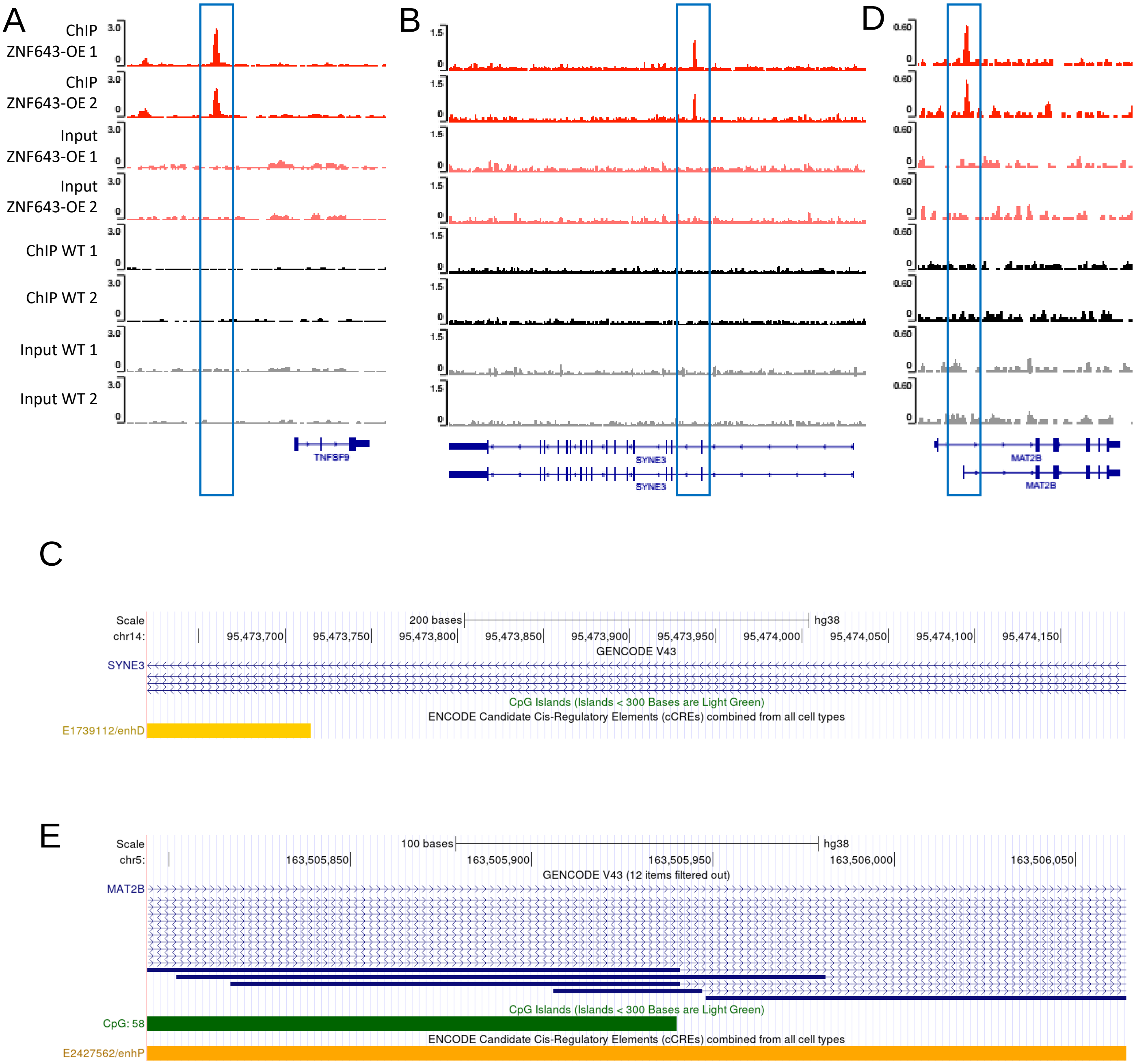

Figure S11. Genomic visualizaion of ZNF643 peaks within specific genomic localizations. Integrative Genomics Viewer (IGV) images of ZNF643 binding regions close to TNFSF9 (A), SYNE3 (B), and MAT2B (D) TSSs. The images show duplicates for HA-ChIP and Input tracks in ZNF643-OE (red/pink colors; upper panel) and WT (black/grey colors; bottom panel) SKMES cel lines. Genome Browser images of ZNF643 binding sites within SYNE3 (C) and MAT2B (E) genes (blue) merged with CpG Island track (green), and ENCODE Candidate Cis-Regulatory Elements (cCREs) combined from all available cell types (yellow) showing distal and proximal (respectively) enhancer regions.
