# Supplementary material for: ZNF643/ZFP69B Exerts Oncogenic Properties and Associates with Cell Adhesion and Immune Processes
Source: Int J Mol Sci. 2023 Nov 15;24(22):16380. doi: 10.3390/ijms242216380 (PMC10671213; doi:10.3390/ijms242216380)
Supplement: Supplementary file 1 [file ijms-24-16380-s001.zip › Supplemental Table S1.pdf]

RNA-seq			
**Gene name**	**Gene symbol**	**Expression in shZNF643 cells**	**Role in ferroptosis**
**H2073**			
Transcription Factor AP-2 Alpha	TFAP2A	upregulated	suppressor
**SKMES**			
Activating Transcription Factor 3	ATF3	downregulated	driver/unclassified
Dual Oxidase 1	DUOX1	downregulated	driver
Interleukin 1 Beta	IL1B	downregulated	driver
MYB Proto-Oncogene, Transcription Factor	MYB	downregulated	driver
Cyclin Dependent Kinase Inhibitor 1A	CDKN1	downregulated	suppressor
Nuclear Receptor Subfamily 4 Group A Member 1	NR4A1	downregulated	suppressor
Nuclear Protein 1, Transcriptional Regulator	NUPR1	upregulated	suppressor
Solute Carrier Family 2 Member 3	SLC2A3	downregulated	unclassified

ChIP-seq				
	**Gene name**	**Peak overlap / Closest TSS**	**Log2foldchange**	**Role in ferroptosis**
ELOVL Fatty Acid Elongase 5	ELOVL5	Overlap symbol	3.02	driver
F-Box And WD Repeat Domain Containing 7	FBXW7	Overlap symbol	1.78	driver
Peroxisome Proliferator Activated Receptor Gamma	PPARG	Overlap symbol	3.52	driver
ZNFX1 Antisense RNA 1	ZFAS1	Overlap symbol	3.61	driver
Dipeptidase 1	DPEP1	Closest TSS	5.53	driver

Supplemental Table S1. Ferroptosis-related genes deregulated or bound by ZNF643. The table represents DEGs identified in shZNF643 lung cell lines (H2073 and SKMES) and ZNF643-bound genes (in SKMES cell line) with known function in ferroptosis.
